# Transcriptional Response of *Wolbachia* to Dengue Virus Infection in Cells of the Mosquito Aedes aegypti

**DOI:** 10.1128/mSphere.00433-21

**Published:** 2021-06-30

**Authors:** Michael Leitner, Cameron Bishop, Sassan Asgari

**Affiliations:** a Australian Infectious Disease Research Centre, School of Biological Sciences, The University of Queensland, Brisbane, Australia; Clemson University

**Keywords:** *Aedes aegypti*, *w*AlbB, *Wolbachia*, dengue virus, mosquito, transcription

## Abstract

Aedes aegypti transmits one of the most significant mosquito‐borne viruses, dengue virus (DENV). The absence of effective vaccines and clinical treatments and the emergence of insecticide resistance in A. aegypti necessitate novel vector control strategies. A new approach uses the endosymbiotic bacterium Wolbachia pipientis to reduce the spread of arboviruses. However, the *Wolbachia*-mediated antiviral mechanism is not well understood. To shed light on this mechanism, we investigated an unexplored aspect of *Wolbachia*-virus-mosquito interaction. We used RNA sequencing to examine the transcriptional response of *Wolbachia* to DENV infection in A. aegypti Aag2 cells transinfected with the *w*AlbB strain of *Wolbachia*. Our results suggest that genes encoding an endoribonuclease (RNase HI), a regulator of sigma 70-dependent gene transcription (6S RNA), essential cellular, transmembrane, and stress response functions and primary type I and IV secretion systems were upregulated, while a number of transport and binding proteins of *Wolbachia,* ribosome structure, and elongation factor-associated genes were downregulated due to DENV infection. Furthermore, bacterial retrotransposon, transposable, and phage-related elements were found among the up- and downregulated genes. We show that *Wolbachia* elicits a transcriptional response to virus infection and identify differentially expressed *Wolbachia* genes mostly at the early stages of virus infection. These findings highlight *Wolbachia*’s ability to alter its gene expression in response to DENV infection of the host cell.

**IMPORTANCE** Aedes aegypti is a vector of several pathogenic viruses, including dengue, Zika, chikungunya, and yellow fever viruses, which are of importance to human health. *Wolbachia* is an endosymbiotic bacterium currently used in transinfected mosquitoes to suppress replication and transmission of dengue viruses. However, the mechanism of *Wolbachia*-mediated virus inhibition is not fully understood. While several studies have shown mosquitoes’ transcriptional responses to dengue virus infection, none have investigated these responses in *Wolbachia*, which may provide clues to the inhibition mechanism. Our results suggest changes in the expression of a number of functionally important *Wolbachia* genes upon dengue virus infection, including those involved in stress responses, providing insights into the endosymbiont’s reaction to virus infection.

## INTRODUCTION

Arthropod-borne viruses (arboviruses) account for a significant number of viral infections in humans and livestock, representing a considerable global public health burden (1, 2). Dengue virus (DENV) is overwhelmingly the most important mosquito-borne flavivirus, responsible for an estimated 390 million infections annually with a further 2.5 billion people at risk of infection worldwide (1, 3). DENV consists of four distinct serotypes (DENV-1 to -4) and is a member of the *Flaviviridae* family that encompasses lipid-enveloped, single-stranded positive-sense RNA viruses (4). The main transmission vectors, the mosquitoes Aedes aegypti and Aedes albopictus, are distributed in high abundance worldwide, especially in regions with tropical and subtropical climates such as Asia, mid-Africa, Central America, and most of South America (5, 6). DENV has fully adapted to a human-mosquito-human transmission cycle, with A. aegypti being the primary vector of DENV in high-density urban environments (7, 8). *A. albopictus* is also capable of transmitting DENV; however, it predominantly colonizes suburban, rural, and forest areas (8). Dramatic increases in population density accompanied by rapid urbanization and cross-linked global transport systems are responsible for the rise of mosquito vectors throughout the tropical regions of the world (1, 7–9).

In light of the absence of an effective vaccine or clinical treatment, sustainable alternatives to control the global spread of DENV are being investigated. Historically, vector control programs against A. aegypti, reliant on chemical and biological agents, have been the methods of choice to reduce DENV transmissions (10). However, with the emergence of insecticide resistance in A. aegypti across many regions of the world, the effectiveness of chemical-based vector control interventions has declined dramatically (10–13). As a result, novel vector control strategies are needed to stop the expansion of mosquito-borne arboviruses.

A new effective approach to reduce the spread of arboviruses such as DENV, Zika virus (ZIKV), and chikungunya virus (CHIKV) uses the endosymbiotic bacterium Wolbachia pipientis as a biological control agent (14–16). It is estimated that various strains of *Wolbachia* infect 40% to 60% of all arthropod species (17–19). *Wolbachia* are Gram-negative, obligate intracellular alphaproteobacteria that have evolved to invade, influence, and manipulate their arthropod host’s reproductive system in order to establish within a host population (18, 20, 21). *Wolbachia*’s ability to proliferate through a population is mainly achieved by inducing cytoplasmic incompatibility (CI) in its host (20, 22). The phenomenon of CI occurs when a *Wolbachia-*infected male insect mates with a female insect that is uninfected or not infected with a compatible strain of *Wolbachia*, generating nonviable offspring (20, 22). Consequently, CI promotes the unaided dispersion of the exclusively maternally inherited *Wolbachia* infection into the host population (20, 22). Additionally, in *Wolbachia-*transinfected female mosquitoes, *Wolbachia* conveys a fitness advantage by protecting the mosquitoes against infectious microbes, including viruses (20, 23, 24). The ability of certain strains of *Wolbachia* to provide protection against RNA virus infections was originally discovered in Drosophila melanogaster by two independent groups of researchers in 2008 (25, 26). The discovery of this insect protection against RNA viruses revealed the association between virus resistance and *Wolbachia* infection, paving the way for an effective mechanism to control mosquito-borne diseases.

*Wolbachia* does not naturally occur in A. aegypti; however, stable transinfections can be achieved through microinjections (27). Several studies in A. aegypti investigating artificially introduced *Wolbachia* strains such as *w*MelPop, *w*Mel, and *w*AlbB have demonstrated *Wolbachia*-induced changes in expression of immune-related genes (15, 28, 29). These findings led the way to two main hypotheses for *Wolbachia*-mediated virus inhibition, that of host innate immune priming and the competition for limited cellular host resources required for virus replication (14, 30). Mosquito species such as A. aegypti depend on their innate immune system to control arbovirus replication, as they lack the adaptive immune system of vertebrates (31). The interaction between the replicating DENV and its host A. aegypti’s innate immune system affects viral replication and the successive transmission of DENV (32). In this regard, a number of studies have found that the innate immune response and mediated pathways such as the RNA interference (RNAi), Toll, immune deficiency (IMD), and JAK/STAT signaling pathways have antiviral effects in A. aegypti (33, 34). Initially, the innate immune response in mosquitoes is activated through the recognition of pathogens by various receptors (35). The RNAi pathway is activated by the detection of double-stranded RNA (dsRNA) molecules of viral genomes or replication intermediates (34). Within the RNAi pathway, two RNA molecules, microRNA (miRNA) and small interfering RNA (siRNA), are vital for the RNAi pathway’s functionality (36). Interestingly, both miRNAs and siRNAs are modified upon *Wolbachia* infections (37). The inhibition of certain miRNAs induces a reduction of *Wolbachia* density, indicating that *Wolbachia* promotes its own preservation within the host (37). Several studies have confirmed that *Wolbachia* infections enhance the basal gene expression of the Toll, JAK/STAT, and RNAi pathways (15, 28, 29, 38). A study conducted in an A. aegypti (Aag2)-derived clonal cell line infected with *Wolbachia* demonstrated upregulation of endonuclease argonaute-2 (Ago2), a core catalytic component within the RNA-induced silencing complex (RISC) of the RNAi pathway (38). The *Wolbachia*-induced upregulation of Ago2 results in increased activity of the RNAi pathway, subsequently providing better protection against arboviruses (38). Overall, these findings have identified the RNAi pathway as the key insect antiviral pathway capable of limiting arboviruses such as DENV, CHIKV, and Sindbis virus (SINV) (31, 32, 39). However, studies suggest that RNAi may play no or a limited role in *Wolbachia*-mediated virus inhibition (38, 40).

Competition for limited cellular host resources also has been subject to investigations (14, 41). In particular, blood meal-derived molecules such as cholesterol are essential in mosquito egg development (41, 42). A. aegypti infected with *Wolbachia* strains *w*MelPop and *w*Mel showed a 15% to 25% reduction in total cholesterol quantities compared to that in uninfected controls (41). However, subsequent cholesterol supplementation administered via blood meal did not correct the altered embryo morphology phenotype, and it did not improve fecundity or egg viability deficits (41). Understanding the contributions of A. aegypti, DENV, and *Wolbachia* in the virus inhibition phenotype is challenging and is further complicated by the fact that *Wolbachia* cannot be genetically modified (30, 43). The majority of knowledge to date has been gained from comparing the durability and level of *Wolbachia*-mediated virus inhibition in different combinations of vector species, virus genotypes, and *Wolbachia* strains (30, 44). Dissecting the mechanism of *Wolbachia*-mediated virus inhibition in A. aegypti proves difficult, and the inhibition is likely multilayered, with no individual mechanism responsible for this phenotype (30, 43). The diverse differences of *Wolbachia* strains infecting the same A. aegypti host confirm the contribution of the symbiont’s genome to the mosquito-*Wolbachia* association (30, 45). Remarkably, the ability and magnitude of *Wolbachia*-mediated antiviral inhibition can differ between *Wolbachia* strains (15, 46–50).

Thus far, the arthropod host A. aegypti response to viral infections has been extensively investigated; however, to our knowledge, the transcriptional response of *Wolbachia* to virus infections in the mosquito has not. For this reason, we examined whether *Wolbachia*’s transcriptional response to DENV infection may shed light on the *Wolbachia*-mediated viral inhibition mechanism based on the function of differentially expressed genes. To test our hypothesis, we compared the transcriptional response of *Wolbachia w*AlbB strain to DENV infection in a transinfected A. aegypti (Aag2)-derived cell line. The outcomes of this study provide a basis for follow-up investigations into the mechanism.

## RESULTS

### RNA-Seq data analysis of Aag2.*w*AlbB cells after infection with DENV.

**FIG 1:**
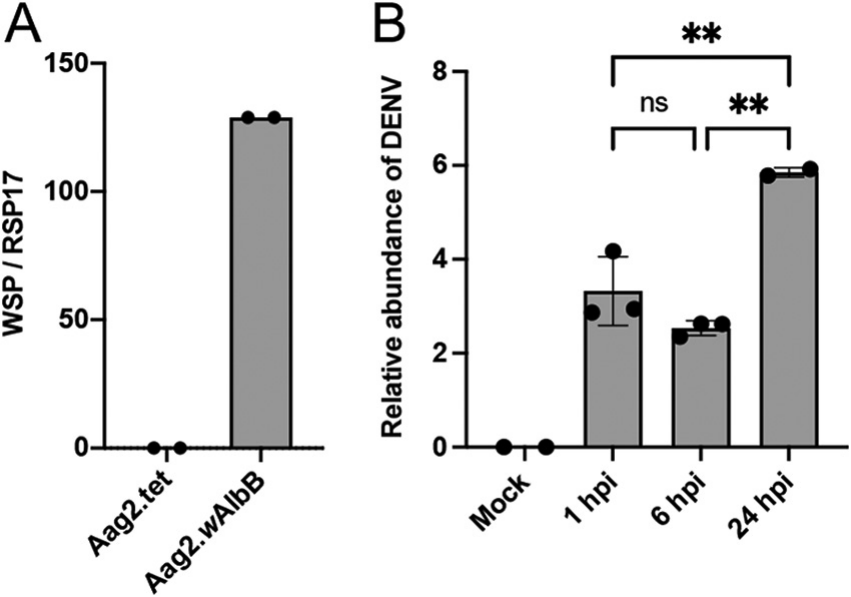
Relative density of *Wolbachia* in Aag2.*w*AlbB cell line and infectivity of the DENV inoculum. (A) The relative *Wolbachia* density was measured by qPCR analysis using genomic DNA extracted from Aag2.*w*AlbB cells and tetracycline-cured *Wolbachia*‐free Aag2.tet cells as a control. Primers targeting the *Wolbachia* surface protein gene (*wsp*) and A. aegypti *RPS17* to amplify the target and normalizing genes were used, respectively. (B) Validation of virus infection by RT-qPCR analysis confirming DENV-2 genome copy accumulation in infected Aag2.*w*AlbB samples used for RNA-Seq. Uninfected Aag2.*w*AlbB cells served as control (mock). Sample 24a-infected was excluded from analysis. The bars and error bars represent means and standard deviations (SDs), respectively, from the three biological replicates. One-way analysis of variance (ANOVA) with Tukey’s *post hoc* multiple-comparison tests were used for data analysis. ns, not significant; **, *P* < 0.01.

To identify differentially expressed genes of *Wolbachia w*AlbB strain in response to DENV infection in transinfected A. aegypti Aag2 cells, DENV-2-infected and uninfected Aag2.*w*AlbB cells were collected at 1, 6, and 24 h postinfection (hpi) in three biological replicates for each time point. We chose these early time points, because previous studies have indicated that *Wolbachia*-mediated virus inhibition is exerted at early hours of infection in insect cells, including mosquito cells (51, 52). Prior to the experiment, *w*AlbB’s relative density was quantified by quantitative PCR (qPCR), which showed amplification of the *wsp* gene in Aag2.*w*AlbB cells, whereas no amplification of the gene was detected in the Aag2.tet cells (Aag2.*w*AlbB cells previously treated with tetracycline to remove *Wolbachia*) (Fig. 1A). *Wolbachia* density is expressed in terms of the number of *wsp* copies relative to the number of the mosquito’s *RPS17* copies. Furthermore, increasing DENV genome copy accumulation was confirmed in the samples at time points 1, 6, and 24 hpi by quantitative reverse transcription-PCR (RT-qPCR) (Fig. 1B). While DENV genome copy numbers did not significantly change between 1 and 6 hpi, a significant increase in genome copy numbers was detected at 24 hpi. Based on a previous study in *A. albopictus* C6/36 cells, accumulation of viral genomic RNA of the four DENV serotypes started from 13 to 18 hpi (53). Therefore, no detectable viral replication between 1 and 6 hpi is expected.

**FIG 2:**
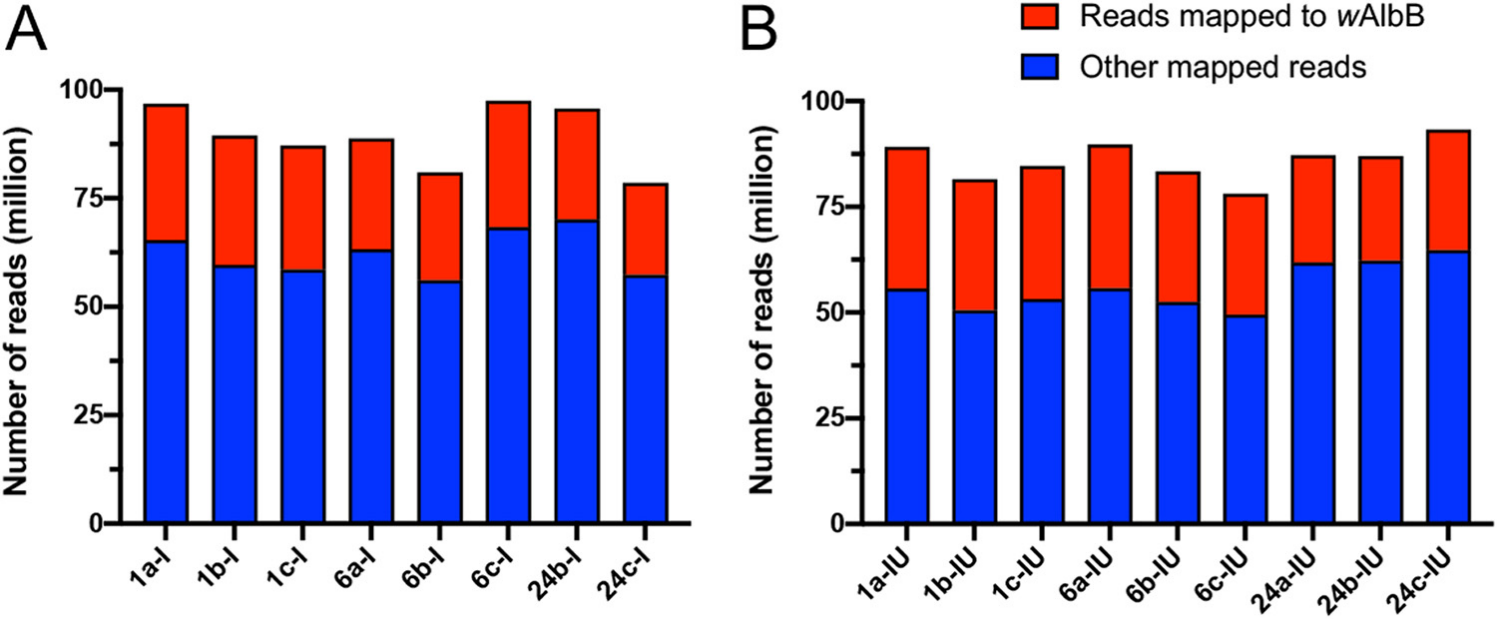
Composition of Aag2.*w*AlbB normalized reads mapped to the combined reference file and the *w*AlbB reference genome. Aag2.*w*AlbB reads were mapped to A. aegypti, *w*AlbB, and viral reference genomes for DENV-infected (A) and uninfected (B) Aag2.*w*AlbB cells. Sample 24a-infected was excluded from analysis. The bars represent total numbers of reads mapped to all the genomes, with the red portion representing reads mapped to the *w*AlbB genome and the blue portion representing reads that did not map to the *w*AlbB genome but to the rest (A. aegypti and viral reference genomes).

**FIG 3:**
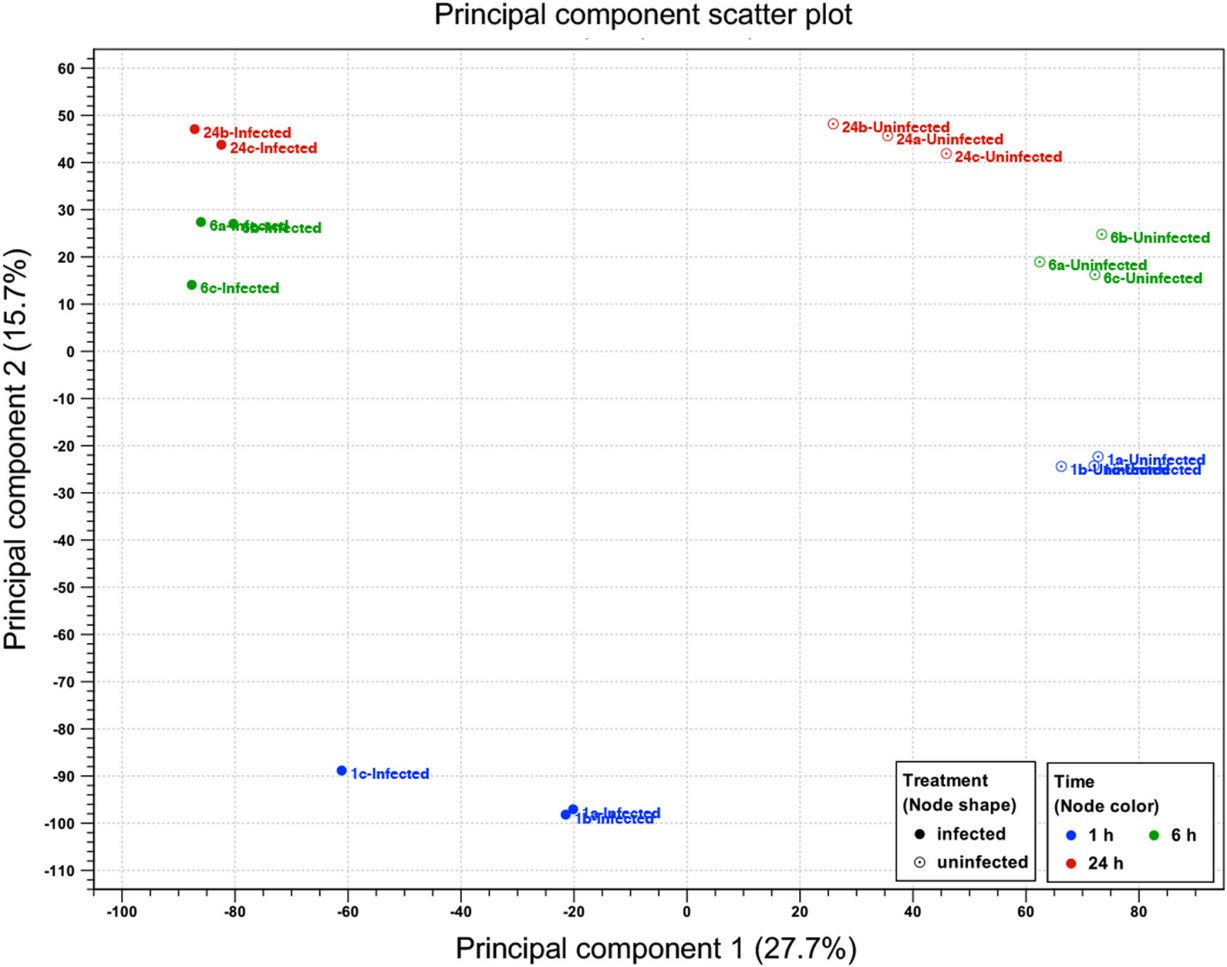
Principal-component analysis (PCA) of the RNA-Seq samples. The PCA figure represents a two-dimensional scatterplot of the first two principal components of the RNA-Seq data. DENV-infected and uninfected Aag2.*w*AlbB sample groups at different time points are represented by different colors as indicated by the legend provided within the graph. Each dot represents a biological replicate of an RNA-Seq sample. Sample 24a-infected was excluded from analysis.

Illumina HiSeq sequencing of RNA from DENV-2-infected and uninfected Aag2.*w*AlbB cells generated a total of 1,629,779,502 raw reads with 150-bp paired-ends from the 18 RNA samples (see Table S2 in the supplemental material). Following the adapter trimming and quality filtering using the trim reads sequence tool in CLC Genomics Workbench (CLC-GWB), a total of 1,629,758,722 high-quality reads remained. Each sample and each biological condition were represented by an average of 92,742,982 and 88,341,320 million reads, respectively. The trimmed reads with a Phred quality score of >Q30 corresponding to an error rate of 0.04% were mapped to the A. aegypti three chromosomes, mitochondrion, *w*AlbB, DENV-2, and Aag2 cell line-related viruses (cell-fusing agent virus [CFAV] and Aedes albopictus negev-like virus [AaNLV]) reference genomes using the RNA sequencing (RNA-Seq) analysis tool in CLC-GWB as described in Materials and Methods. An average of 90,044,894 and 86,018,515 reads mapped to the A. aegypti, *w*AlbB, and virus genomes in the DENV-infected and uninfected samples, respectively, equivalent to 97.4% of the total reads (Fig. 2). Sample 24a infected with DENV-2 was excluded from further analysis, as we found that RNA from Aag2.tet cells at 24 hpi was accidently submitted for sequencing instead of Aag2.*w*AlbB at 24 hpi. However, the data were used for *de novo* assembly of the DENV-2 ET-300 genome (see below). For each sample and biological condition, averages of 27,005,569 and 29,785,073 reads for DENV-infected and uninfected samples, respectively, equivalent to 29.1% to 33.7%, were mapped to the *w*AlbB’s reference genome (54). Principal-component analysis (PCA) analysis showed distinct separation at 1, 6, and 24 hpi between DENV-infected and uninfected Aag2.*w*AlbB samples, indicating a high level of similarity among the biological replicates from different samples (Fig. 3). The 2.6% unmapped reads were used for *de novo* assembly of contigs using the CLC-GWB *de novo* assembly tool. BLASTn analysis of the assembled contigs to the nonredundant nucleotide NCBI DNA database produced a variety of long and short contigs. The longest contigs between 1,801 and 13,186 bp in size were identified as additional A. aegypti host-related viruses, Aedes anphevirus, Primus virus, Phasi Charoen-like virus, Aedes albopictus densovirus, Australian Anopheles totivirus, and unplaced scaffolds of A. aegypti genome artifacts (55).

10.1128/mSphere.00433-21.5 TABLE S2 Information relevant to the RNA-Seq data. Download Table S2, XLSX file, 0.1 MB. Copyright © 2021 Leitner et al. 2021 Leitner et al. https://creativecommons.org/licenses/by/4.0/ This content is distributed under the terms of the Creative Commons Attribution 4.0 International license.

**FIG 4:**
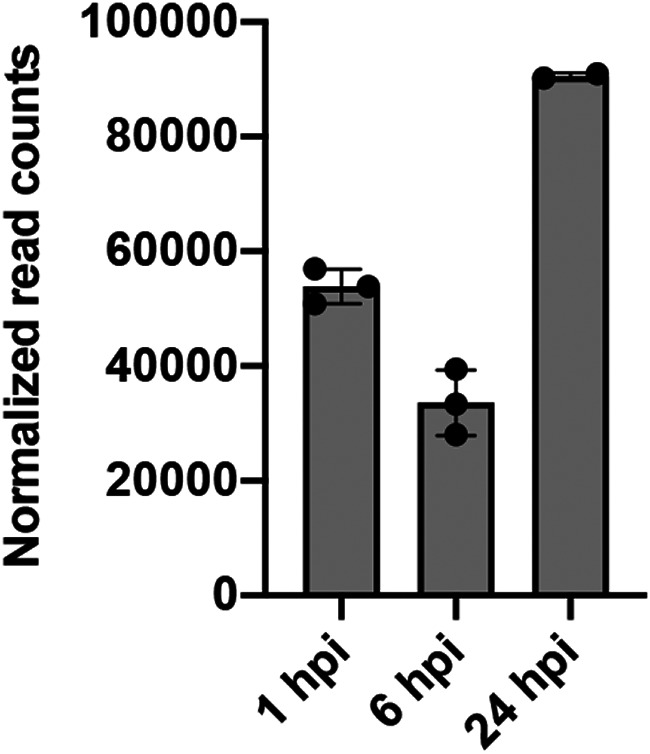
Accumulation of DENV-2 read counts following infection in RNA-Seq samples. Validation of virus infection: full-length virus genome read counts expressed in normalized read counts (TMM adjusted) confirmed DENV abundance in each infected Aag2.*w*AlbB sample used for RNA-Seq. Sample 24a-infected was excluded from analysis. The bars and error bars represent means and SDs from the three biological replicates.

Full-length virus genome read counts expressed in normalized read counts (trimmed mean of M values [TMM] adjusted) confirmed the increasing DENV abundance in each DENV-infected Aag2.*w*AlbB sample used for RNA-Seq (Fig. 4).

### Transcriptional response by *Wolbachia* with respect to time and DENV infection.

**FIG 5:**
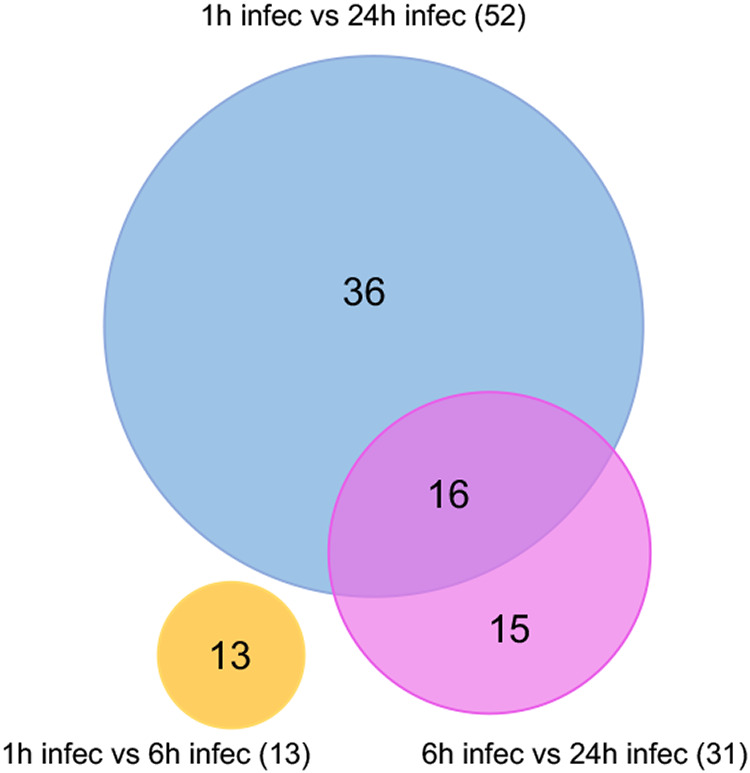
Differentially expressed genes in *w*AlbB across time points after DENV infection. Venn diagram showing DEGs (fold change of ≥2.0 and an adjusted *P* value of <0.05) in *w*AlbB in DENV-infected Aag2.*w*AlbB mosquito cells when different time points were compared. Sixteen overlapping DEGs were found between the time points. Each colored circle represents a sample collection time point.

A total of 96 genes were significantly differentially expressed across the three time points combined; 13, 31, and 52 differentially expressed genes (DEGs) at 1 versus 6, 6 versus 24, and 1 versus 24 hpi, respectively (Fig. 5; see also Table S3). There was a total of 16 DEGs that overlapped between the time points (Fig. 5). The open reading frames (ORFs) from the identified DEGs featured complete coding with no premature stop codon truncations. Approximately one-quarter of those DEGs, 26.0% (25), were upregulated versus 74.0% (56) being significantly downregulated. Specifically, 4, 13, and 8 genes were upregulated at 1 versus 6, 6 versus 24, and 1 versus 24 hpi in contrast to 9, 18, and 44 downregulated genes at the three time points, respectively (Table S3).

10.1128/mSphere.00433-21.6 TABLE S3 Differentially expressed *Wolbachia w*AlbB genes upon DENV infection over time. Download Table S3, XLSX file, 0.1 MB. Copyright © 2021 Leitner et al. 2021 Leitner et al. https://creativecommons.org/licenses/by/4.0/ This content is distributed under the terms of the Creative Commons Attribution 4.0 International license.

Among the upregulated genes at 1 versus 6 hpi were those encoding transposases, conjugal transfer protein TraJ from the type IV secretion system, and MSF transporter. Downregulated genes identified in that comparison were those encoding an ATPase, ankyrin repeat domain-containing protein, RNase HI, ATP synthase subunit C, and five hypothetical proteins (Table S3). DEGs, when 6 and 24 hpi samples were compared, represented transposases, ankyrin repeat domain-containing proteins, RuvC, *O*-methyltransferase, a number of ribosomal proteins, maturase, ferredoxin, MspII family outer membrane protein, elongation factor Tu, a reductase, TrbC from the type IV secretory system, iron-sulfur cluster assembly accessory protein, sodium:proton antiporter, and a number of hypothetical proteins (Table S3). When data from 1 versus 24 hpi were compared, similar types of genes were differentially expressed, including those encoding transposases, *O*-methyltransferase, maturase, epimerase, ribosomal proteins, ion antiporters, ATPases, elongation factor Tu, and ankyrin repeat domain-containing proteins plus a number of other proteins that are all listed in Table S3.

### Transcriptional response by *Wolbachia* to DENV infection.

**FIG 6:**
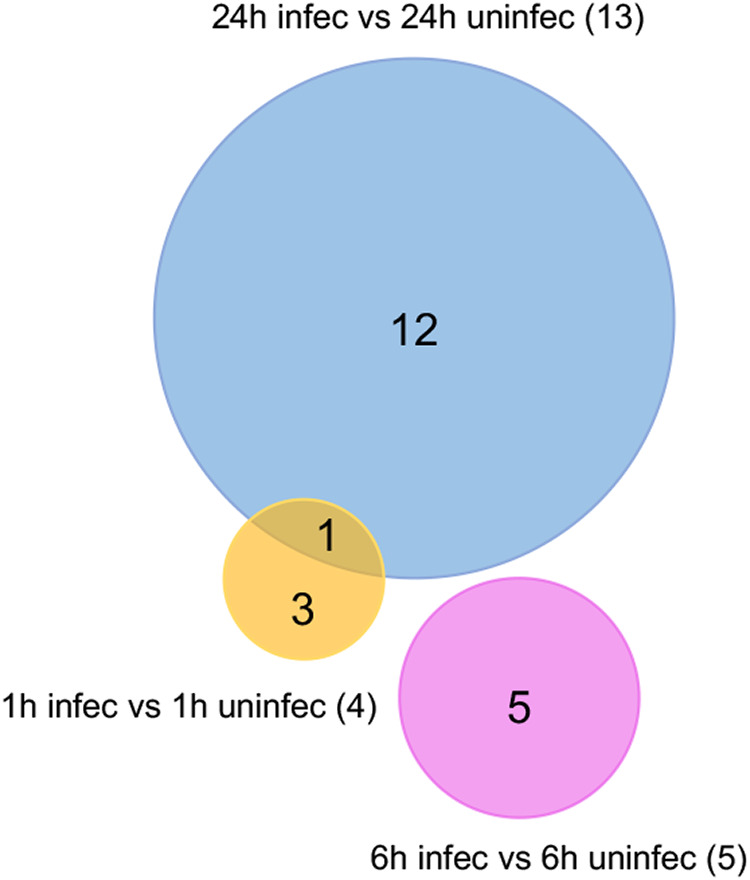
Differentially expressed genes in *w*AlbB due to DENV infection. Venn diagram showing DEGs (fold change of ≥2.0 and an adjusted *P* value of <0.05) in *w*AlbB in DENV-infected versus uninfected Aag2.*w*AlbB mosquito cells at each time point. One overlap of DEGs was found between the time points. Each colored circle represents a sample collection time point.

When DENV-infected and uninfected samples at each time point were compared, a total of 22 genes were significantly differentially expressed across the three time points combined, considering a fold change of ≥2.0 and an adjusted *P* value of <0.05: 4, 5, and 13 DEGs at 1, 6, and 24 hpi, respectively (Fig. 6; see also Table S4). These included genes encoding DNA mismatch repair protein MutS, RNase HI, and transposases at 1 hpi, glutathione synthase, group II intron reverse transcriptase, and transposase at 6 hpi and *O*-methyltransferase, SPFH domain-containing protein, group II intron reverse transcriptases, sodium:proton antiporter, ribosomal proteins, and iron-sulfur cluster assembly accessory protein at 24 hpi (Table S4). There was only one DEG that overlapped between the time points, encoding DNA mismatch repair protein MutS (Fig. 6).

10.1128/mSphere.00433-21.7 TABLE S4 Differentially expressed *Wolbachia w*AlbB genes upon DENV infection at each time point. Download Table S4, XLSX file, 0.1 MB. Copyright © 2021 Leitner et al. 2021 Leitner et al. https://creativecommons.org/licenses/by/4.0/ This content is distributed under the terms of the Creative Commons Attribution 4.0 International license.

However, considering a fold change of ≥1.5, a total of 148 genes were significantly differentially expressed across the three time points combined: 39, 65, and 44 DEGs at 1, 6, and 24 hpi, respectively (Fig. S2; Table S4). There was a total of 11 overlapping DEGs between the time points (Fig. S2). These included genes encoding MutS, ATP-binding protein, DnaJ, two group II intron reverse transcriptases, ankyrin domain-containing protein, TatD hydrolase, cytidylyltransferase, ribosomal protein S9, and a hypothetical protein. A third of those DEGs, 25.0% (37), were upregulated versus 75.0% (57) being significantly downregulated. Specifically, 21, 1, and 15 genes were upregulated at 1, 6, and 24 hpi in contrast to 18, 64, and 29 downregulated genes at the three time points, respectively (Table S4).

10.1128/mSphere.00433-21.2 FIG S2 Differentially expressed genes in *w*AlbB due to DENV infection. Venn diagram showing DEGs (fold-change of ≥1.5 and an adjusted *P* value of <0.05) in *w*AlbB in DENV-infected versus uninfected Aag2.*w*AlbB cells at each time point. Eleven overlapping DEGs were found between the time points. Each colored circle represents a sample collection time point. Download FIG S2, TIF file, 0.1 MB. Copyright © 2021 Leitner et al. 2021 Leitner et al. https://creativecommons.org/licenses/by/4.0/ This content is distributed under the terms of the Creative Commons Attribution 4.0 International license.

### *De novo* assembly of DENV-2 ET-300 genome.

Despite the usage of DENV-2 ET-300 strain in several studies, the complete genome of the virus was not available on GenBank at the time this study took place. To assemble the viral genome, we used reads from Aag2.*w*AlbB sample 24a. In Aag2.*w*AlbB sample 24a, 678,422 of 100,812,616 (0.67%) reads mapped to a 10,696-nucleotide long contig, achieving an average coverage of 9,476×. The largest ORF from this contig showed complete coding with no premature stop codon truncations present. The ORF is 10,176 nucleotides in length and encodes a 3,392-amino-acid polyprotein. BLASTn analysis identified the ORF as 99.28% identical with the highest pairwise nucleotide identity (10,091/10,164) to dengue virus 2 genomic RNA, strain D2/Hu/OPD030NIID/2005 (GenBank identifier [ID] LC111438.1; E value, 0.0; query cover, 99%). BLASTp analysis of the ORF revealed that it is most similar to the polyprotein of dengue virus 2 (GenBank ID BAU36333.1; E value, 0.0; identity, 99.71%; query cover, 100%) and contains the three classic structural (C, prM, and E) and seven nonstructural (NS1, NS2A, NS2B, NS3, NS4A, NS4B, and NS5) proteins of the DENV polyprotein (58–60). The complete genome sequence obtained in this study is 100% identical to a recently deposited sequence for DENV-2 ET-300 (accession number MT921572).

### Validation of DEGs by quantitative reverse transcription-PCR.

**FIG 7:**
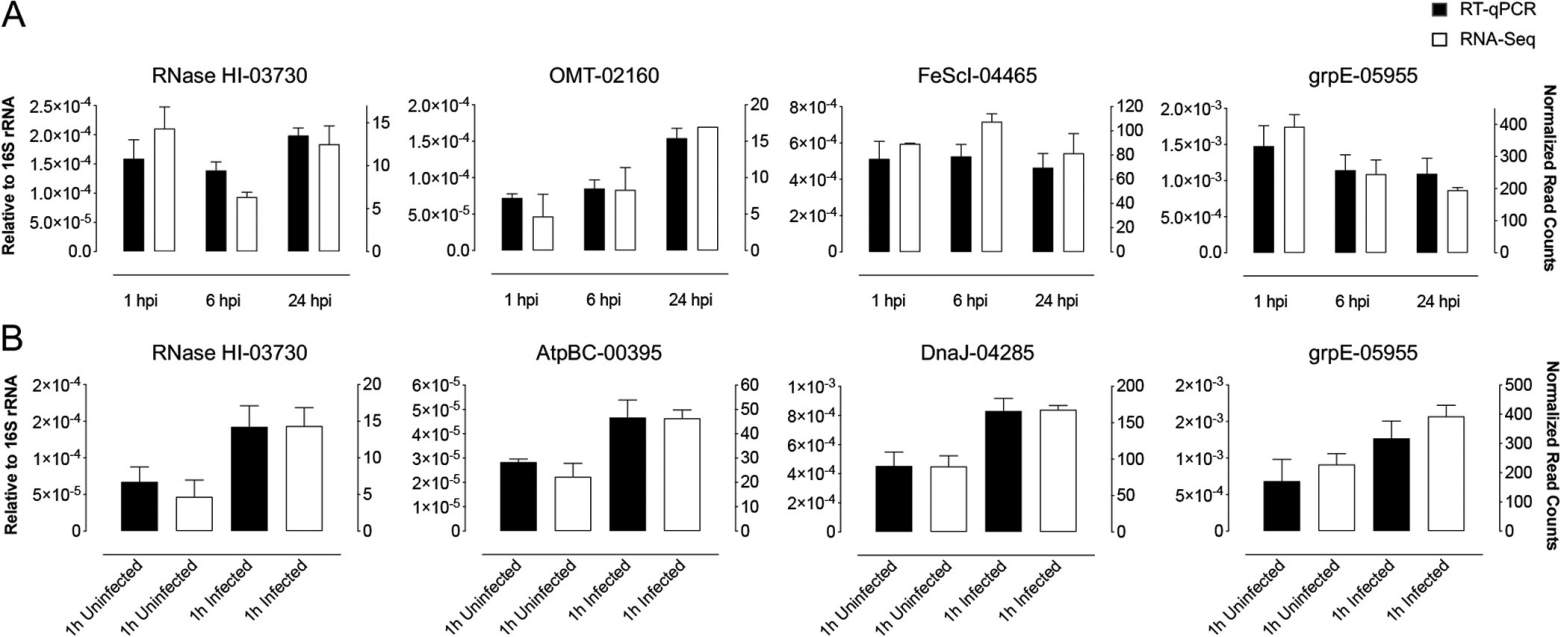
Validation of differentially expressed *Wolbachia* genes after DENV-2 infection using RT-qPCR. (A) The bar graphs represent RNA-Seq normalized gene reads and RT-qPCR relative expression results of the DEGs in DENV-infected Aag2.*w*AlbB cells with respect to time and DENV infection. (B) The bar graphs represent RNA-Seq normalized gene reads and RT-qPCR relative expression results of the DEGs in DENV-infected versus uninfected Aag2.*w*AlbB cells at 1 hpi.

The representative selection of differentially expressed *Wolbachia* genes identified by RNA-Seq analysis was validated by RT-qPCR. For this, the DENV infection experiment was repeated in Aag2.*w*AlbB cells with identical conditions to the preparation of samples for RNA-Seq. DENV infection was confirmed in cells collected at 1, 6, and 24 hpi (see Fig. S3). The results showed an overall consistency between RNA-Seq and RT-qPCR, when DEGs in DENV-infected Aag2.*w*AlbB cells with respect to time and DENV infection were considered (Fig. 7A). Similarly, we found consistency between the two approaches when DENV-infected and uninfected samples were compared (Fig. 7B).

10.1128/mSphere.00433-21.3 FIG S3 Confirmation of DENV infection in Aag2.*w*AlbB cells. (A) RT-qPCR analysis confirmed DENV-2 genome copy accumulation in infected Aag2.*w*AlbB samples used for validation of RNA-Seq data by RT-qPCR. Uninfected Aag2.*w*AlbB cells served as a control (mock). The bars and error bars represent means and SDs from two biological replicates. Download FIG S3, TIF file, 0.1 MB. Copyright © 2021 Leitner et al. 2021 Leitner et al. https://creativecommons.org/licenses/by/4.0/ This content is distributed under the terms of the Creative Commons Attribution 4.0 International license.

## DISCUSSION

The endosymbiotic bacterium *Wolbachia* has proven to be an effective biocontrol strategy to reduce the transmission of arboviruses by mosquitoes. A number of studies have confirmed that *Wolbachia* confers antiviral protection in primary arbovirus mosquito vectors (14–16, 61). Nonetheless, the mechanism underlying the *Wolbachia*-mediated viral protection is not yet well understood. The mosquito host A. aegypti response to viral infections has been extensively investigated (56, 62, 63); however, to our knowledge, the transcriptional response of *Wolbachia* to virus infections in the mosquito has not. For this reason, we examined whether *Wolbachia*’s transcriptional response to DENV infection could shed light on the *Wolbachia*-mediated viral inhibition mechanism based on the function of differentially expressed genes. We used RNA-Seq in order to study the transcriptional response of the *Wolbachia w*AlbB strain to DENV-2 infection in A. aegypti Aag2 cells.

We found an exponential increase of the transcriptional changes of *Wolbachia* with respect to time and DENV infection occurring between 1 and 6 hpi (13 DEGs), followed by 6 and 24 hpi (31 DEGs), with most changes between 1 and 24 hpi (52 DEGs). Similarly, the transcriptional response by *Wolbachia* to DENV infection when comparing DENV-infected versus uninfected Aag2.*w*AlbB cells at 1, 6, and 24 hpi showed increases in transcriptional changes across the three time points for 4, 5, and 13 genes, respectively. At 1 hpi, we identified a gene encoding an endoribonuclease (RNase HI) as significantly upregulated due to DENV infection. RNase HI plays an essential role in bacterial cellular processes such as DNA replication, gene expression, DNA repair, and degradation of RNA (64–66) and is an endoribonuclease with the capability of degrading RNA/DNA hybrids generated during viral replication in a non-sequence-specific manner (64, 66, 67). Furthermore, studies have suggested that RNase HI could play a vital role in antiviral defense in prokaryotes (68–71).

Using a fold change difference of ≥1.5 provided a more complete insight into *Wolbachia*’s transcriptional response to DENV infection and uncovered a number of significant differentially expressed genes related to stress response, secretion, and fundamental bacterial cellular functions based on annotation. Among the upregulated genes at 1 hpi were genes encoding the DNA mismatch repair protein MutS, molecular chaperone DnaJ, nucleotide exchange factor GrpE, ATP-binding cassette (ABC) domain-containing protein, and type I (HlyD family periplasmic adaptor subunit) and IV (conjugative DNA transfer family protein VirD4 and P-type conjugative transfer protein VirB9) secretory system elements. *mutS* encodes an essential DNA mismatch repair protein, MutS, that identifies and corrects errors generated during DNA replication and other biological processes (72, 73). The heat shock molecular chaperone protein-encoding genes *dnaJ* and *grpE* participate in responses to heat shock and cellular stress, stabilizing proteins by preventing their denaturation and actively refolding denatured proteins (74–78). Both *dnaJ* and *grpE* are part of the major DnaK/DnaJ chaperone system used by prokaryotic symbionts in response to stress (78, 79). The nucleotide exchange factor GrpE functions as a molecular cochaperone promoting the exchange of ADP for ATP from DnaK within the bacterial cytoplasm (75, 76). ABC domain-containing proteins are part of the ABC transmembrane transport system involved in the import of essential nutrients and the export of various molecules such as proteins, lipids, and toxins (80–82). In *Wolbachia*, ABC transmembrane transporters are embedded in the inner membrane and usually connected to a membrane fusion protein that crosses the periplasmic space (83). The type I secretion system (T1SS) is a basic secretion system spanning both the inner and outer membranes of *Wolbachia* (83–85). This enables the transport of substrates (e.g., DNA and proteins) from the cytoplasm to outside the cell into the host (83–85). The T1SS is composed of three proteins: an ABC transporter/ATPase (ABC) embedded in the inner membrane, a membrane fusion protein, and an outer membrane protein (83, 85). The T4SS protein VirD4 is an important ATPase known as a coupling protein responsible for substrate recruitment within the T4SS (86, 87). The T4SS transports substrates (e.g., DNA and proteins) from the cytoplasm to outside the cell or into various target recipients, including eukaryotic cells, and spans both membranes and the periplasm (83, 87–89). Essentially, all members of the *Rickettsiales* harbor T1SS and T4SS implicated in their establishment, pathogenicity, and transmission (83). Despite all of these genes being significantly upregulated or inducted at the very early time point of DENV infection, they were all downregulated over time, i.e., at 24 hpi.

Among the downregulated genes at 1 hpi is an RNase (RNAP), a DNA-dependent RNA polymerase known as the key enzyme in gene expression and regulation in bacteria (90–92). Despite the fact that the gene encoding the RNA polymerase was downregulated at 1 hpi, it was upregulated over time, and the mean gene expression across all biological replicates and time points remained very high. Furthermore, two ankyrin repeat-containing (ANK) proteins and a site-specific recombinase were identified as WO prophage-related genes (54, 93, 94). ANK (DEJ70_02765) and the site-specific recombinase (DEJ70_04945) are located within the *w*AlbB WO-like island 01 and *w*AlbB WO-like island 02, respectively. ANK proteins are rare in most bacteria; however, 34 *w*AlbB proteins contain at the minimum one copy of an ANK repeat domain, with a total of 81 copies of various types of ANK repeat domains (54). More than 30 *Wolbachia* strains from different insect and nematode hosts feature these ANK repeat regions (83, 94–96). These ANK proteins mediate a wide range of protein-protein interactions, such as transcription initiation and signaling, and are believed to be involved in *Wolbachia*-host interactions (54, 94–97).

At 6 hpi, a regulator of sigma 70-dependent gene transcription *ssrS* (6S RNA), a highly abundant noncoding RNA (ncRNA), was the only DEG upregulated, whereas all 64 of the other DEGs were downregulated (98–102). Concurrently, DENV genome copy numbers decreased notably at 6 hpi. Studies in Escherichia coli and other bacteria have shown that 6S RNA specifically binds σ70 RNA polymerase capable of inhibiting and downregulating transcription at σ70-dependent promoters (98–102). Furthermore, ncRNAs such as 6S RNA have been identified to perform several essential functions such as RNA processing, gene regulation, and interaction with proteins in addition to mRNAs (57, 98–102). In *Wolbachia* strains, *w*Mel, *w*MelPop-CLA, *w*MelCS, and *w*Au studies discovered a range of ncRNAs and confirmed their biological functionality (57, 103). However, both studies failed to detect 6S RNA in the *Wolbachia* strains used. Mayoral et al. used Illumina TruSeq small RNA deep sequencing and detecting read fragments of 40 bp in size (103), whereas Woolfit et al. produced sequence fragments of 300 bp, acknowledging that this size is longer than most known small RNAs (sRNAs) (57). Eminently, 6S RNA in *w*AlbB is 162 bp in size, containing conserved prokaryotic promoter regions and boxes −35 and −10 indicative of its functionality to regulate bacterial and even host gene transcription, as previously described (103).

At the later time point, 24 hpi, the majority of DEGs were related to ribosomal structure, translation, DNA mismatch repair, transmembrane, and stress response. Furthermore, a number of secretion system subunit-encoding genes were downregulated due to DENV infection over time. Bacterial transposable elements and a number of hypothetical proteins were found among the upregulated and downregulated genes across all time points. IS*3*, and IS*66* family transposase are insertion sequences (IS) which comprise the simplest form of transposable elements (TEs) of a transposase gene, flanked by inverted repeats (54, 104, 105). IS elements comprise 13% of the *w*AlbB genome, corresponding to a total number of 218 different IS element families (54).

Furthermore, 16 overlapping DEGs were identified between 6- and 24-hpi and 1- and 24-hpi time points. Most of these genes are related to basic bacterial cellular functions and TEs, such as amino acids, isoprenoid biosynthesis, methyltransferase, ribosomes, group II intron bacterial retrotransposon, and transposase.

The functional importance of the differentially expressed genes in response to DENV infection across the three different time points potentially points toward a transcriptional response by *Wolbachia* to DENV infection and the associated cellular stress experienced. *Wolbachia* elicits the upregulation of RNase HI, an endoribonuclease capable of degrading RNA-DNA hybrids generated during viral replication in a non-sequence-specific manner, due to DENV infection at 1 hpi (64, 66, 67). This is followed by a stress response using the major DnaK/DnaJ chaperone system (*dnaJ* and *grpE* upregulated due to DENV infection at 1 hpi), which is used by prokaryotic symbionts in response to thermal stress (54, 78, 79), while concurrently upregulating genes associated with T1SS and T4SS transmembrane transport functions, known to facilitate the translocation of effector proteins such as ANKs involved in *Wolbachia*-host interactions (54, 94–97). Despite the upregulation of the major DnaK/DnaJ chaperone system and secretion pathway subunits at the very early stage of DENV infection, they were downregulated over time. Remarkably, the majority of DEGs at 6 hpi were downregulated, while 6S RNA, a ncRNA and known negative regulator of sigma 70-dependent gene transcription (98–102), was upregulated.

Unlike what we found in mosquito cells, a couple of studies on *Drosophila* did not find major transcriptional changes in *Wolbachia* following virus infection. In one study, the investigators combined a *Wolbachia*-infected Drosophila melanogaster cell line JW18 with the mosquito-borne Semliki Forest virus (SFV; alphavirus) to examine the mechanism of antiviral protection (51). RNA-Seq was performed on samples collected at 7 and 24 hpi; however, Rainey et al. reported a lack of differentially expressed *Wolbachia* genes between cells infected with and without SFV (51). Similarly, when D. melanogaster flies harboring the *w*Mel2 strain of *Wolbachia* were infected with Sindbis virus (an alphavirus), no transcriptional changes in the *w*Mel2 transcriptome were observed at 6, 24, and 48 h postinjection (106). The discrepancy between our findings and the two studies in D. melanogaster cell lines and flies could be due to differences in the *Wolbachia* strains, host cells, and the viruses used in the studies.

In conclusion, our findings shed new light on *Wolbachia*’s transcriptional response to DENV infection of the host cell and identify differentially expressed genes starting as early as 1 hpi, which to our knowledge, have not been examined so far. Further research, especially into RNase HI, 6S RNA, and other proteins and signaling molecules which are involved in *Wolbachia*’s response to stress and virus infection is necessary to uncover their potential contribution to antiviral protection. Furthermore, considering *Wolbachia* and cytoplasmic RNA viruses compete for resources, changes in the expression of genes that affect the availability of energy and resources to the virus, imposed by *Wolbachia*, could contribute to reduction in virus propagation. This line of research is, however, currently hampered due to the unavailability of tools to genetically modify or silence *Wolbachia* genes.

## MATERIALS AND METHODS

### Mosquito cell line maintenance.

An Aag2.*w*AlbB cell line was developed previously (107) by transinfecting Aag2 cells with *w*AlbB from Aa23 cells. Aa23 cells infected with *w*AlbB were established from eggs of Aedes albopictus (108). Cells were maintained in 1:1 Mitsuhashi-Maramorosch (Himedia) and Schneider’s Drosophila medium (Invitrogen) supplemented with 10% fetal bovine serum (FBS; Bovogen Biologicals, France) at 27°C and passaged every 3 to 4 days.

### Virus infection experiments.

Prior to the infection of Aag2.*w*AlbB cells, *Wolbachia* density was quantified by quantitative PCR (qPCR). Genomic DNA was extracted from cells using the DIY spin column protocol as previously described (109). qPCR was performed using, QuantiFast SYBR green PCR mix (Qiagen), in accordance with the manufacturer’s instructions. Forward and reverse primers (*w*AlbB-*wsp*-qF and *w*AlbB*-wsp*-qR) (see Table S1 in the supplemental material) targeting the *Wolbachia* surface protein gene (*wsp*) and A. aegypti ribosomal protein subunit 17 gene (*RPS17*) (AeRPS17-qF and AeRPS17-qR) (Table S1) were used to amplify the target and the normalizing gene, respectively (14, 37). All qPCRs were performed in duplicates using a Rotor-Gene Q thermocycler (Qiagen) under the following conditions: 95°C for 5 min and then 40 cycles of 95°C for 10 s and combined annealing/extension at 60°C for 35 s. This was followed by a standard post-qPCR high-resolution melt analysis to assess the specificity of the amplified *wsp* and *RPS17* qPCR products. The relative abundance of *wsp* and *RPS17* was determined using the relative quantification method as described previously (110).

10.1128/mSphere.00433-21.4 TABLE S1 Primers used in this study. Download Table S1, PDF file, 0.1 MB. Copyright © 2021 Leitner et al. 2021 Leitner et al. https://creativecommons.org/licenses/by/4.0/ This content is distributed under the terms of the Creative Commons Attribution 4.0 International license.

For virus infection, Aag2.*w*AlbB cells were seeded into 6-well culture plates (Greiner Bio-One) at 1.5 × 10^6^ cells per well and allowed to adhere for 1 h at 27°C. Medium was then removed, and cells were inoculated with DENV serotype 2 (DENV-2) East Timor strain (ET-300) at a multiplicity of infection (MOI) of 1. Uninfected cells were treated the same as Aag2.*w*AlbB virus-infected cells except without the addition of virus. All 6-well plates were incubated on a rocker for 1 h at room temperature followed by the removal of supernatant and subsequent replenishment with fresh medium. The plates were then incubated at 27°C. Aag2.*w*AlbB virus-infected and uninfected cells were collected at 1, 6, and 24 h postinfection (hpi) in three biological replicates for each time point. In our experimental design, we used matched time points for infected and uninfected samples to take into consideration possible changes of gene expression over time in both *Wolbachia* and host cells in cell culture. Cells were pelleted by centrifugation at 2,500 × *g* for 3.5 min. Supernatant was discarded and pellets were frozen at −80°C for future RNA extraction.

Leftover RNA samples were used to confirm DENV-2 infection (see below for RNA extraction). For quantitation of DENV-2 genome copies in Aag2.*w*AlbB cells, first-strand cDNA synthesis was performed using M-MuLV reverse transcriptase (New England BioLabs) with a gene-specific (NS5) reverse primer (DENV2-qR) (Table S1) to amplify the DENV genomic RNA and A. aegypti *RPS17* reverse primer (AeRPS17-qR) (Table S1) for the synthesis of the reference gene cDNA. In each RT reaction, 500 ng of DNase I-treated RNA was used as the template in a total volume of 20 μl. All the RT reactions were performed in a Veriti 96-well thermocycler (Applied Biosystems) under the following conditions: 65°C for 5 min, 50°C for 50 min, and 75°C for 15 min. For qPCR, the produced cDNA was diluted in a 1:5 ratio with Ultrapure DNase/RNase-free water (Invitrogen). Two microliters of the diluted cDNA was used for qPCRs with both forward and reverse gene-specific primers (DENV2-qF and DENV2-qR) (Table S1) used to amplify the DENV‐2 NS5 region of the viral genome (111). A. aegypti *RPS17* forward and reverse primers were used to amplify the normalizing gene (14). All qPCRs were performed in duplicates using a Rotor-Gene Q thermocycler (Qiagen) under the conditions specified above.

### RNA extraction and sequencing.

Total RNA from DENV-2-infected and uninfected Aag2.*w*AlbB cells was extracted using the Qiagen RNeasy minikit (Qiagen) according to the manufacturer’s instructions. The obtained RNA pellets were resuspended in 30 μl of Ultrapure DNase/RNase-free water (Invitrogen) and treated with DNase I enzyme (TURBO DNase; Invitrogen) to remove genomic DNA from RNA samples as per the manufacturer’s instructions. RNA sequencing (RNA-Seq) was conducted by GENEWIZ (GENEWIZ, China) under the following conditions. The RNA quality and quantity of each sample were assessed using a Bioanalyzer 2100 (Agilent Technologies), Agilent RNA 6000 Nano kit (Agilent Technologies), and 1% agarose gel. In total, 5 μg of total RNA per sample with an RNA integrity number of >7.0 was used for library preparation according to the manufacturer’s protocol using NEBNext Ultra RNA library prep kit for Illumina (New England BioLabs). After removal of ribosomal RNAs using a Ribo-Zero-rRNA removal kit (Illumina), the mRNA fragmentation and priming were performed using NEBNext first-strand synthesis reaction buffer and NEBNext random primers (New England BioLabs). First-strand cDNA was synthesized using ProtoScript II reverse transcriptase, and the second-strand cDNA was synthesized using a second-strand synthesis enzyme mix (New England BioLabs). The double-stranded DNA was purified by AxyPrep Mag PCR clean-up (Axygen) and treated with End Prep enzyme mix to repair both ends and add deoxyribosyladenine (dA) tailing in one reaction, followed by a T-A ligation to add adaptors to both ends. Size selection of adaptor-ligated DNA was then performed using AxyPrep Mag PCR clean-up (Axygen), and fragments of ∼360 bp (with the approximate insert size of 300 bp) were recovered. Each sample was then amplified by PCR using P5 and P7 primers (Table S1). The PCR products were cleaned up using AxyPrep Mag PCR clean-up (Axygen), validated using an Agilent 2100 Bioanalyzer (Agilent Technologies), and quantified by a Qubit 2.0 fluorometer (Invitrogen). Then, libraries with different indices were multiplexed and loaded on an Illumina HiSeq instrument according to the manufacturer’s instructions (Illumina). Sequencing was carried out using a 2 by 150-bp paired-end (PE) configuration, and image analysis and base calling were conducted by the HiSeq Control Software (HCS)+OLB+GAPipeline-1.6 on the Illumina HiSeq instrument (Illumina). The sequences were processed and quality controlled by GENEWIZ.

Data quality control using demultiplexing was performed by bcl2fastq 2.17, and RNA-Seq raw data were filtered as follows. Low-quality pair-end reads with ambiguous nucleotide content of N bases of more than 10% and a ratio of bases of low quality (Phred quality score Q < 20) of more than 0.5 in either read were filtered out and discarded before data analysis. The paired-end raw reads were obtained as fastq files and then imported into CLC Genomics Workbench v20.0.2 (CLC-GWB; Qiagen) for further processing and analysis. The raw reads were trimmed from the 3′ ends using the trim reads sequence tool in CLC-GWB in order to remove Illumina sequencing adapter P5 and P7. Default trim settings with automatic read-through adapter trimming and removal of low-quality sequences (Phred quality score = 0.05) were applied.

### Differential gene expression analysis.

Aag2.*w*AlbB high-quality trimmed paired-end reads were mapped to a combined reference genome assembly containing the A. aegypti strain LVP_AGWG AaegL5.0 chromosomes 1 to 3 (NC_035107, NC_035108, and NC_035109), mitochondrion complete genome (MF194022), *Wolbachia w*AlbB genome (CP031221), cell-fusing agent virus strain Galveston genome (CFAV; NC_001564), Aedes albopictus negev-like virus isolate (AaNLV; MK879802), and the *de novo* assembled DENV-2 ET-300 genome (see below for assembly). It was previously shown that Aag2.*w*AlbB cells are persistently infected with CFAV and AalNLV (112). The mapping setting “genome annotated with genes and transcripts suitable for Eukaryotes” was used, which accounts for splicing. Mapping parameters with a minimum length fraction of 0.8, minimum similarity fraction of 0.9, and maximum number of hits for a read of 30 to capture multimapping repetitive elements were applied. The CLC-GWB principal-component analysis (PCA) for RNA-Seq tool was used to create a two-dimensional principal component plot to identify outlying samples and variations in the sample data set.

The empirical analysis of differentially expressed genes (DEGs) was performed using the CLC-GWB differential expression for RNA-Seq tool with the default parameters to identify DEGs between DENV-infected and uninfected Aag2.*w*AlbB samples. TMM (trimmed mean of M values) normalization was used to calculate effective library sizes as developed and described previously (113). The CLC-GWB differential expression for RNA-Seq tool uses multifactorial statistics based on a negative binomial generalized linear model (GLM) for this purpose (114). Differential gene expression in response to DENV infection was evaluated at 1, 6, and 24 hpi between virus-infected and uninfected samples, whereas the differential expression with respect to time and DENV infection was assessed between 1 versus 6, 6 versus 24, and 1 versus 24 hpi (Fig. S1). In the latter comparisons, up- or downregulation of a gene was considered over time. For both sets of analyses, we used CLC-GWB’s “all group pairs” analysis, which tests for differences between all pairs of groups in a factor, including a Benjamini-Hochberg correction for multiple comparisons, and accordingly adjusts *P* values. Significance of DEGs was assessed using the Wald test, and results were then filtered based on a fold change of ≥2.0, ≥1.5, and adjusted *P* value of <0.05. The CLC-GWB create Venn diagram for RNA-Seq tool was used to compare and visualize the overlap of DEGs between the three sampling time points. Open reading frame (ORF) integrity of identified DEGs was assessed using the Swiss Institute of Bioinformatics (SIB) ExPasy protein analysis tool. A summary of the procedures from read mapping to the analysis of *w*AlbB differentially expressed genes in response to DENV infection is shown in Fig. S1.

10.1128/mSphere.00433-21.1 FIG S1 The flow chart shows the steps taken for the analysis of the RNA-Seq data followed by the analysis of *w*AlbB’s differentially expressed genes (DEGs) with respect to time and infection with dengue virus. X, Y, and Z, and their variations represent the number of differentially expressed genes between two conditions. Download FIG S1, TIF file, 0.2 MB. Copyright © 2021 Leitner et al. 2021 Leitner et al. https://creativecommons.org/licenses/by/4.0/ This content is distributed under the terms of the Creative Commons Attribution 4.0 International license.

For validation of DENV infection, normalized read counts mapping to the virus genome were used to estimate DENV abundance in each sample used for RNA-Seq.

### *De novo* assembly of DENV-2 ET-300.

After adapter and quality trimming of Aag2.*w*AlbB, clean reads were *de novo* assembled using CLC-GWB *de novo* assembly tool with the default parameters for word length, bubble, and minimum contig length of 200 nucleotides. Assembled contig reads were mapped back to contigs with stringent mapping settings (mismatch cost, 2; insertion cost, 3; deletion cost, 3; length fraction, 0.8; similarity fraction, 0.8) to eliminate false-positive mapping. CLC-GWB find ORFs tool with default settings was used to identify ORFs within the assembled contig. The assembled contig was then queried using NCBI’s BLASTn (BLASTn v.2.10.1+) nonredundant nucleotide and BLASTp (BLASTp v.2.10.1+) nonredundant protein sequence databases.

### Validation of DEGs using quantitative reverse transcription-PCR.

For validation of the differentially expressed *Wolbachia* genes determined through RNA-Seq analysis, a total of 8 up- and downregulated genes were selected. The NCBI Primer-BLAST primer design tool was used to design primers for the selected DEGs and the 16S rRNA normalizing gene (115). The primers are listed in Table S1. New RNA samples were generated by repeating the infection experiment with DENV-2 ET-300 under identical conditions as before by collecting virus-infected and uninfected Aag2.*w*AlbB cells at 1, 6, and 24 hpi. RNA extraction, reverse transcription, and qPCR were performed as described above, except that first-strand cDNA synthesis was performed using SuperScript III reverse transcription (Invitrogen) and random hexamer primers (New England BioLabs) to synthesize a cDNA strand from extracted RNA as per the manufacturer’s instructions. No reverse transcriptase control reactions were also included in the RT-qPCRs. *Wolbachia*’s 16S rRNA gene was used as the normalizing gene. The relative abundance of 16S rRNA and selected DEGs was determined using the relative quantification method as described previously (110).

### Data availability.

The RNA-Seq data generated in this study were submitted to SRA under the project ID PRJNA669319.

## References

[1] Bhatt S, Gething P, Brady O, Messina J, Farlow A, Moyes C, Drake J, Brownstein J, Hoen A, Sankoh O, Myers M, George D, Jaenisch T, Wint G, Simmons C, Scott T, Farrar J, Hay S. 2013. The global distribution and burden of dengue. Nature 496:504–507. doi:10.1038/nature12060. 23563266 PMC3651993

[2] Hill CA, Kafatos FC, Stansfield SK, Collins FH. 2005. Arthropod-borne diseases: vector control in the genomics era. Nat Rev Microbiol 3:262–268. doi:10.1038/nrmicro1101. 15703759

[3] Guzman MG, Halstead SB, Artsob H, Buchy P, Farrar J, Gubler DJ, Hunsperger E, Kroeger A, Margolis HS, Martinez E, Nathan MB, Pelegrino JL, Simmons C, Yoksan S, Peeling RW. 2010. Dengue: a continuing global threat. Nat Rev Microbiol 8:S7–S16. doi:10.1038/nrmicro2460. 21079655 PMC4333201

[4] Acosta EG, Kumar A, Bartenschlager R. 2014. Revisiting dengue virus–host cell interaction: new insights into molecular and cellular virology. Adv Virus Res 88:1–109. doi:10.1016/B978-0-12-800098-4.00001-5. 24373310

[5] Kraemer MUG, Sinka ME, Duda KA, Mylne A, Shearer FM, Brady OJ, Messina JP, Barker CM, Moore CG, Carvalho RG, Coelho GE, Van Bortel W, Hendrickx G, Schaffner F, Wint GRW, Elyazar IRF, Teng H-J, Hay SI. 2015. The global compendium of *Aedes aegypti* and *A. albopictus* occurrence. Sci Data 2:150035. doi:10.1038/sdata.2015.35. 26175912 PMC4493829

[6] Liu-Helmersson J, Brännström Å, Sewe MO, Semenza JC, Rocklöv J. 2019. Estimating past, present, and future trends in the global distribution and abundance of the arbovirus vector *Aedes aegypti* under climate change scenarios. Front Public Health 7:148. doi:10.3389/fpubh.2019.00148. 31249824 PMC6582658

[7] Gubler DJ. 2011. Dengue, urbanization and globalization: the unholy trinity of the 21(st) century. Trop Med Health 39:3–11. doi:10.2149/tmh.2011-S05. PMC3317603 22500131

[8] Gould E, Pettersson J, Higgs S, Charrel R, de Lamballerie X. 2017. Emerging arboviruses: why today? One Health 4:1–13. doi:10.1016/j.onehlt.2017.06.001. 28785601 PMC5501887

[9] Eder M, Cortes F, Teixeira de Siqueira Filha N, Araújo de França GV, Degroote S, Braga C, Ridde V, Turchi Martelli CM. 2018. Scoping review on vector-borne diseases in urban areas: transmission dynamics, vectorial capacity and co-infection. Infect Dis Poverty 7:90. doi:10.1186/s40249-018-0475-7. 30173661 PMC6120094

[10] Marcombe S, Mathieu RB, Pocquet N, Riaz M-A, Poupardin R, Sélior S, Darriet F, Reynaud S, Yébakima A, Corbel V, David J-P, Chandre F. 2012. Insecticide resistance in the dengue vector *Aedes aegypti* from Martinique: distribution, mechanisms and relations with environmental factors. PLoS One 7:e30989. doi:10.1371/journal.pone.0030989. 22363529 PMC3283601

[11] Marcombe S, Carron A, Darriet F, Etienne M, Agnew P, Tolosa M, Yp-Tcha MM, Lagneau C, Yébakima A, Corbel V. 2009. Reduced efficacy of pyrethroid space sprays for dengue control in an area of Martinique with pyrethroid resistance. Am J Trop Med Hygiene 80:745–751. doi:10.4269/ajtmh.2009.80.745. 19407118

[12] Bellinato DF, Viana-Medeiros PF, Araujo SC, Martins AJ, Lima JB, Valle D. 2016. Resistance status to the insecticides temephos, deltamethrin, and diflubenzuron in Brazilian *Aedes aegypti* populations. BioMed Res Int 2016:8603263. doi:10.1155/2016/8603263. 27419140 PMC4932163

[13] Moyes C, Vontas J, Martins A, Ng LC, Koou S, Dusfour I, Raghavendra K, Pinto J, Corbel V, David JP, Weetman D. 2017. Contemporary status of insecticide resistance in the major *Aedes* vectors of arboviruses infecting humans. PLoS Negl Trop Dis 11:e0005625. doi:10.1371/journal.pntd.0005625. 28727779 PMC5518996

[14] Moreira LA, Iturbe-Ormaetxe I, Jeffery JA, Lu G, Pyke AT, Hedges LM, Rocha BC, Hall-Mendelin S, Day A, Riegler M, Hugo LE, Johnson KN, Kay BH, McGraw EA, van Den Hurk AF, Ryan PA, O'Neill SL. 2009. A *Wolbachia* symbiont in *Aedes aegypti* limits infection with dengue, chikungunya, and *Plasmodium*. Cell 139:1268–1278. doi:10.1016/j.cell.2009.11.042. 20064373

[15] Bian G, Xu Y, Lu P, Xie Y, Xi Z. 2010. The endosymbiotic bacterium *Wolbachia* induces resistance to dengue virus in *Aedes aegypti* (*Wolbachia*-dengue interactions). PLoS Pathog 6:e1000833. doi:10.1371/journal.ppat.1000833. 20368968 PMC2848556

[16] Walker T, Johnson PH, Moreira LA, Iturbe-Ormaetxe I, Frentiu FD, McMeniman CJ, Leong YS, Dong Y, Axford J, Kriesner P, Lloyd AL, Ritchie SA, O'Neill SL, Hoffmann AA. 2011. The *w*Mel *Wolbachia* strain blocks dengue and invades caged *Aedes aegypti* populations. Nature 476:450–453. doi:10.1038/nature10355. 21866159

[17] Rasgon JL, Gamston CE, Ren X. 2006. Survival of *Wolbachia pipientis* in cell-free medium. Appl Environ Microbiol 72:6934–6937. doi:10.1128/AEM.01673-06. 16950898 PMC1636208

[18] Zug R, Hammerstein P. 2012. Still a host of hosts for *Wolbachia*: analysis of recent data suggests that 40% of terrestrial arthropod species are infected. PLoS One 7:e38544. doi:10.1371/journal.pone.0038544. 22685581 PMC3369835

[19] de Oliveira CD, Gonçalves DS, Baton LA, Shimabukuro PHF, Carvalho FD, Moreira LA. 2015. Broader prevalence of *Wolbachia* in insects including potential human disease vectors. Bull Entomol Res 105:305–315. doi:10.1017/S0007485315000085. 25772521

[20] Dobson SL, Marsland EJ, Rattanadechakul W. 2002. Mutualistic *Wolbachia* infection in *Aedes albopictus*: accelerating cytoplasmic drive. Genetics 160:1087–1094. doi:10.1093/genetics/160.3.1087. 11901124 PMC1462033

[21] McGraw EA, O'Neill SL. 2004. *Wolbachia pipientis*: intracellular infection and pathogenesis in *Drosophila*. Curr Opin Microbiol 7:67–70. doi:10.1016/j.mib.2003.12.003. 15036143

[22] Werren JH, Baldo L, Clark ME. 2008. *Wolbachia*: master manipulators of invertebrate biology. Nat Rev Microbiol 6:741–751. doi:10.1038/nrmicro1969. 18794912

[23] Weeks AR, Turelli M, Harcombe WR, Reynolds KT, Hoffmann AA. 2007. From parasite to mutualist: rapid evolution of *Wolbachia* in natural populations of *Drosophila*. PLoS Biol 5:e114. doi:10.1371/journal.pbio.0050114. 17439303 PMC1852586

[24] Jiggins FM. 2017. The spread of *Wolbachia* through mosquito populations. PLoS Biol 15:e2002780. doi:10.1371/journal.pbio.2002780. 28570608 PMC5453404

[25] Hedges LM, Brownlie JC, O'Neill SL, Johnson KN. 2008. *Wolbachia* and virus protection in insects. Science 322:702. doi:10.1126/science.1162418. 18974344

[26] Teixeira L, Ferreira A, Ashburner M. 2008. The bacterial symbiont *Wolbachia* induces resistance to RNA viral infections in *Drosophila melanogaster*. PLoS Biol 6:e1000002. doi:10.1371/journal.pbio.1000002. PMC2605931 19222304

[27] McMeniman CJ, Lane RV, Cass BN, Fong AW, Sidhu M, Wang YF, O'Neill SL. 2009. Stable introduction of a life-shortening *Wolbachia* infection into the mosquito *Aedes aegypti*. Science 323:141–144. doi:10.1126/science.1165326. 19119237

[28] Kambris Z, Cook PE, Phuc HK, Sinkins SP. 2009. Immune activation by life-shortening *Wolbachia* and reduced filarial competence in mosquitoes. Science 326:134–136. doi:10.1126/science.1177531. 19797660 PMC2867033

[29] Rancès E, Ye YH, Woolfit M, McGraw EA, O'Neill SL. 2012. The relative importance of innate immune priming in *Wolbachia*-mediated dengue interference. PLoS Pathog 8:e1002548. doi:10.1371/journal.ppat.1002548. 22383881 PMC3285598

[30] Terradas G, McGraw EA. 2017. *Wolbachia*-mediated virus blocking in the mosquito vector *Aedes aegypti*. Curr Opin Insect Sci 22:37–44. doi:10.1016/j.cois.2017.05.005. 28805637

[31] McFarlane M, Arias-Goeta C, Martin E, O'Hara Z, Lulla A, Mousson L, Rainey SM, Misbah S, Schnettler E, Donald CL, Merits A, Kohl A, Failloux A-B. 2014. Characterization of *Aedes aegypti* innate-immune pathways that limit chikungunya virus replication. PLoS Negl Trop Dis 8:e2994. doi:10.1371/journal.pntd.0002994. 25058001 PMC4109886

[32] Sanchez-Vargas I, Scott JC, Poole-Smith BK, Franz AW, Barbosa-Solomieu V, Wilusz J, Olson KE, Blair CD. 2009. Dengue virus type 2 infections of *Aedes aegypti* are modulated by the mosquito's RNA interference pathway. PLoS Pathog 5:e1000299. doi:10.1371/journal.ppat.1000299. 19214215 PMC2633610

[33] Fragkoudis R, Attarzadeh-Yazdi G, Nash AA, Fazakerley JK, Kohl A. 2009. Advances in dissecting mosquito innate immune responses to arbovirus infection. J Gen Virol 90:2061–2072. doi:10.1099/vir.0.013201-0. 19570957

[34] Blair CD. 2011. Mosquito RNAi is the major innate immune pathway controlling arbovirus infection and transmission. Future Microbiol 6:265–277. doi:10.2217/fmb.11.11. 21449839 PMC3126673

[35] Sim S, Jupatanakul N, Dimopoulos G. 2014. Mosquito immunity against arboviruses. Viruses 6:4479–4504. doi:10.3390/v6114479. 25415198 PMC4246235

[36] Hussain M, Etebari K, Asgari S. 2016. Functions of small RNAs in mosquitoes. Adv Insect Physiol 51:189–222. doi:10.1016/bs.aiip.2016.04.001.

[37] Hussain M, Frentiu FD, Moreira LA, O'Neill SL, Asgari S. 2011. *Wolbachia* uses host microRNAs to manipulate host gene expression and facilitate colonization of the dengue vector *Aedes aegypti*. Proc Natl Acad Sci USA 108:9250–9255. doi:10.1073/pnas.1105469108. 21576469 PMC3107320

[38] Terradas G, Joubert DA, McGraw EA. 2017. The RNAi pathway plays a small part in *Wolbachia*-mediated blocking of dengue virus in mosquito cells. Sci Rep 7:43847. doi:10.1038/srep43847. 28262718 PMC5338330

[39] Campbell CL, Keene KM, Brackney DE, Olson KE, Blair CD, Wilusz J, Foy BD. 2008. *Aedes aegypti* uses RNA interference in defense against Sindbis virus infection. BMC Microbiol 8:47. doi:10.1186/1471-2180-8-47. 18366655 PMC2278134

[40] Hedges LM, Yamada R, O'Neill SL, Johnson KN. 2012. The small interfering RNA pathway is not essential for *Wolbachia*-mediated antiviral protection in *Drosophila melanogaster*. Appl Environ Microbiol 78:6773–6776. doi:10.1128/AEM.01650-12. 22798369 PMC3426675

[41] Caragata EP, Rances E, O'Neill SL, McGraw EA. 2014. Competition for amino acids between *Wolbachia* and the mosquito host, *Aedes aegypti*. Microb Ecol 67:205–218. doi:10.1007/s00248-013-0339-4. 24337107

[42] Ziegler R, Ibrahim MM. 2001. Formation of lipid reserves in fat body and eggs of the yellow fever mosquito, *Aedes aegypti*. J Insect Physiol 47:623–627. doi:10.1016/s0022-1910(00)00158-x. 11249951

[43] Lindsey A, Bhattacharya T, Newton I, Hardy RW. 2018. Conflict in the intracellular lives of endosymbionts and viruses: a mechanistic look at *Wolbachia*-mediated pathogen-blocking. Viruses 10:141. doi:10.3390/v10040141. PMC5923435 29561780

[44] Johnson K. 2015. The impact of *Wolbachia* on virus infection in mosquitoes. Viruses 7:5705–5717. doi:10.3390/v7112903. 26556361 PMC4664976

[45] Terradas G, Allen SL, Chenoweth SF, McGraw EA. 2017. Family level variation in *Wolbachia*-mediated dengue virus blocking in *Aedes aegypti*. Parasit Vectors 10:622. doi:10.1186/s13071-017-2589-3. 29282144 PMC5746003

[46] Lu P, Bian G, Pan X, Xi Z. 2012. *Wolbachia* induces density-dependent inhibition to dengue virus in mosquito cells. PLoS Negl Trop Dis 6:e1754. doi:10.1371/journal.pntd.0001754. 22848774 PMC3404113

[47] Mousson L, Zouache K, Arias-Goeta C, Raquin V, Mavingui P, Failloux A-B. 2012. The native *Wolbachia* symbionts limit transmission of dengue virus in *Aedes albopictus*. PLoS Negl Trop Dis 6:e1989. doi:10.1371/journal.pntd.0001989. 23301109 PMC3531523

[48] Bian G, Zhou G, Lu P, Xi Z. 2013. Replacing a native *Wolbachia* with a novel strain results in an increase in endosymbiont load and resistance to dengue virus in a mosquito vector. PLoS Negl Trop Dis 7:e2250. doi:10.1371/journal.pntd.0002250. 23755311 PMC3675004

[49] Chouin-Carneiro T, Ant TH, Herd C, Louis F, Failloux AB, Sinkins SP. 2020. *Wolbachia* strain *w*AlbA blocks Zika virus transmission in *Aedes aegypti*. Med Vet Entomol 34:116–119. doi:10.1111/mve.12384. 31120156 PMC7027442

[50] Fraser JE, O'Donnell TB, Duyvestyn JM, O'Neill SL, Simmons CP, Flores HA. 2020. Novel phenotype of *Wolbachia* strain *w*Pip in *Aedes aegypti* challenges assumptions on mechanisms of *Wolbachia*-mediated dengue virus inhibition. PLoS Pathog 16:e1008410. doi:10.1371/journal.ppat.1008410. 32726353 PMC7416964

[51] Rainey SM, Martinez J, McFarlane M, Juneja P, Sarkies P, Lulla A, Schnettler E, Varjak M, Merits A, Miska EA, Jiggins FM, Kohl A. 2016. *Wolbachia* blocks viral genome replication early in infection without a transcriptional response by the endosymbiont or host small RNA pathways. PLoS Pathog 12:e1005536. doi:10.1371/journal.ppat.1005536. 27089431 PMC4835223

[52] Bhattacharya T, Newton ILG, Hardy RW. 2020. Viral RNA is a target for *Wolbachia*-mediated pathogen blocking. PLoS Pathog 16:e1008513. doi:10.1371/journal.ppat.1008513. 32555677 PMC7326284

[53] Sakoonwatanyoo P, Boonsanay V, Smith DR. 2006. Growth and production of the dengue virus in C6/36 cells and identification of a laminin-binding protein as a candidate serotype 3 and 4 receptor protein. Intervirology 49:161–172. doi:10.1159/000089377. 16428892

[54] Sinha A, Li Z, Sun L, Carlow CKS. 2019. Complete genome sequence of the *Wolbachia w*AlbB endosymbiont of *Aedes albopictus*. Genome Biol Evol 11:706–720. doi:10.1093/gbe/evz025. 30715337 PMC6414309

[55] Matthews BJ, Dudchenko O, Kingan SB, Koren S, Antoshechkin I, Crawford JE, Glassford WJ, Herre M, Redmond SN, Rose NH, Weedall GD, Wu Y, Batra SS, Brito-Sierra CA, Buckingham SD, Campbell CL, Chan S, Cox E, Evans BR, Fansiri T, Filipović I, Fontaine A, Gloria-Soria A, Hall R, Joardar VS, Jones AK, Kay RGG, Kodali VK, Lee J, Lycett GJ, Mitchell SN, Muehling J, Murphy MR, Omer AD, Partridge FA, Peluso P, Aiden AP, Ramasamy V, Rašić G, Roy S, Saavedra-Rodriguez K, Sharan S, Sharma A, Smith ML, Turner J, Weakley AM, Zhao Z, Akbari OS, Black WC, IV, Cao H, et al. 2018. Improved reference genome of *Aedes aegypti* informs arbovirus vector control. Nature 563:501–507. doi:10.1038/s41586-018-0692-z. 30429615 PMC6421076

[56] Etebari K, Hegde S, Saldaña MA, Widen SG, Wood TG, Asgari S, Hughes GL. 2017. Global transcriptome analysis of *Aedes aegypti* mosquitoes in response to Zika virus infection. mSphere 2:e00456-17. doi:10.1128/mSphere.00456-17. 29202041 PMC5700376

[57] Woolfit M, Algama M, Keith JM, McGraw EA, Popovici J. 2015. Discovery of putative small non-coding RNAs from the obligate intracellular bacterium *Wolbachia pipientis*. PLoS One 10:e0118595. doi:10.1371/journal.pone.0118595. 25739023 PMC4349823

[58] Chambers TJ, Hahn CS, Galler R, Rice CM. 1990. Flavivirus genome organization, expression, and replication. Annu Rev Microbiol 44:649–688. doi:10.1146/annurev.mi.44.100190.003245. 2174669

[59] Gebhard LG, Filomatori CV, Gamarnik AV. 2011. Functional RNA elements in the dengue virus genome. Viruses 3:1739–1756. doi:10.3390/v3091739. 21994804 PMC3187688

[60] Dwivedi VD, Tripathi IP, Tripathi RC, Bharadwaj S, Mishra SK. 2017. Genomics, proteomics and evolution of dengue virus. Brief Funct Genomics 16:217–227. doi:10.1093/bfgp/elw040. 28073742

[61] Hussain M, Lu G, Torres S, Edmonds JH, Kay BH, Khromykh AA, Asgari S. 2013. Effect of *Wolbachia* on replication of West Nile virus in a mosquito cell line and adult mosquitoes. J Virol 87:851–858. doi:10.1128/JVI.01837-12. 23115298 PMC3554047

[62] Colpitts TM, Cox J, Vanlandingham DL, Feitosa FM, Cheng G, Kurscheid S, Wang P, Krishnan MN, Higgs S, Fikrig E. 2011. Alterations in the *Aedes aegypti* transcriptome during infection with West Nile, dengue and yellow fever viruses. PLoS Pathog 7:e1002189. doi:10.1371/journal.ppat.1002189. 21909258 PMC3164632

[63] Bonizzoni M, Dunn WA, Campbell CL, Olson KE, Marinotti O, James AA. 2012. Complex modulation of the *Aedes aegypti* transcriptome in response to dengue virus infection. PLoS One 7:e50512. doi:10.1371/journal.pone.0050512. 23209765 PMC3507784

[64] Cerritelli SM, Crouch RJ. 2009. Ribonuclease H: the enzymes in eukaryotes. FEBS J 276:1494–1505. doi:10.1111/j.1742-4658.2009.06908.x. 19228196 PMC2746905

[65] Majorek KA, Dunin-Horkawicz S, Steczkiewicz K, Muszewska A, Nowotny M, Ginalski K, Bujnicki JM. 2014. The RNase H-like superfamily: new members, comparative structural analysis and evolutionary classification. Nucleic Acids Res 42:4160–4179. doi:10.1093/nar/gkt1414. 24464998 PMC3985635

[66] Tannous E, Kanaya E, Kanaya S. 2015. Role of RNase H1 in DNA repair: removal of single ribonucleotide misincorporated into DNA in collaboration with RNase H2. Sci Rep 5:9969. doi:10.1038/srep09969. 25951507 PMC4423430

[67] Hartmann G. 2017. Nucleic acid immunity. Adv Immunol 133:121–169. doi:10.1016/bs.ai.2016.11.001. 28215278 PMC7112058

[68] Anupama K, Leela JK, Gowrishankar J. 2011. Two pathways for RNase E action in *Escherichia coli in vivo* and bypass of its essentiality in mutants defective for Rho-dependent transcription termination. Mol Microbiol 82:1330–1348. doi:10.1111/j.1365-2958.2011.07895.x. 22026368

[69] Koonin EV. 2017. Evolution of RNA- and DNA-guided antivirus defense systems in prokaryotes and eukaryotes: common ancestry vs convergence. Biol Direct 12:5. doi:10.1186/s13062-017-0177-2. 28187792 PMC5303251

[70] Laalami S, Zig L, Putzer H. 2014. Initiation of mRNA decay in bacteria. Cell Mol Life Sci 71:1799–1828. doi:10.1007/s00018-013-1472-4. 24064983 PMC3997798

[71] Moelling K, Broecker F, Russo G, Sunagawa S. 2017. RNase H as gene modifier, driver of evolution and antiviral defense. Front Microbiol 8:1745. doi:10.3389/fmicb.2017.01745. 28959243 PMC5603734

[72] Fukui K, Kuramitsu S. 2011. Structure and function of the small MutS-related domain. Mol Biol Int 2011:691735. doi:10.4061/2011/691735. 22091410 PMC3200294

[73] Qiu R, Sakato M, Sacho EJ, Wilkins H, Zhang X, Modrich P, Hingorani MM, Erie DA, Weninger KR. 2015. MutL traps MutS at a DNA mismatch. Proc Natl Acad Sci U S A 112:10914–10919. doi:10.1073/pnas.1505655112. 26283381 PMC4568282

[74] Delaney JM. 1990. A grpE mutant of *Escherichia coli* is more resistant to heat than the wild-type. J Gen Microbiol 136:797–801. doi:10.1099/00221287-136-5-797. 2166131

[75] Harrison CJ, Hayer-Hartl M, Di Liberto M, Hartl F, Kuriyan J. 1997. Crystal structure of the nucleotide exchange factor GrpE bound to the ATPase domain of the molecular chaperone DnaK. Science 276:431–435. doi:10.1126/science.276.5311.431. 9103205

[76] Harrison C. 2003. GrpE, a nucleotide exchange factor for DnaK. Cell Stress Chaperones 8:218–224. doi:10.1379/1466-1268(2003)008<0218:GANEFF>2.0.CO;2. 14984054 PMC514874

[77] Aguilar-Rodríguez J, Sabater-Muñoz B, Montagud-Martínez R, Berlanga V, Alvarez-Ponce D, Wagner A, Fares MA. 2016. The molecular chaperone DnaK is a source of mutational robustness. Genome Biol Evol 8:2979–2991. doi:10.1093/gbe/evw176. 27497316 PMC5630943

[78] Roma JS, D'Souza S, Somers PJ, Cabo LF, Farsin R, Aksoy S, Runyen-Janecky LJ, Weiss BL. 2019. Thermal stress responses of *Sodalis glossinidius*, an indigenous bacterial symbiont of hematophagous tsetse flies. PLoS Negl Trop Dis 13:e0007464. doi:10.1371/journal.pntd.0007464. 31738754 PMC6887450

[79] Bhandari V, Houry WA. 2015. Substrate interaction networks of the *Escherichia coli* chaperones: trigger factor, DnaK and GroEL. Adv Exp Med Biol 883:271–294. doi:10.1007/978-3-319-23603-2_15. 26621473

[80] Ren Q, Paulsen IT. 2005. Comparative analyses of fundamental differences in membrane transport capabilities in prokaryotes and eukaryotes. PLoS Comput Biol 1:e27. doi:10.1371/journal.pcbi.0010027. 16118665 PMC1188273

[81] Atkins HS, Dassa E, Walker NJ, Griffin KF, Harland DN, Taylor RR, Duffield ML, Titball RW. 2006. The identification and evaluation of ATP binding cassette systems in the intracellular bacterium *Francisella tularensis*. Res Microbiol 157:593–604. doi:10.1016/j.resmic.2005.12.004. 16503121

[82] Davidson AL, Dassa E, Orelle C, Chen J. 2008. Structure, function, and evolution of bacterial ATP-binding cassette systems. Microbiol Mol Biol Rev 72:317–364. doi:10.1128/MMBR.00031-07. 18535149 PMC2415747

[83] Lindsey ARI. 2020. Sensing, signaling, and secretion: a review and analysis of systems for regulating host interaction in *Wolbachia*. Genes 11:813. doi:10.3390/genes11070813. PMC7397232 32708808

[84] Bing XL, Zhao DS, Sun JT, Zhang KJ, Hong XY. 2020. Genomic analysis of *Wolbachia* from *Laodelphax striatellus* (Delphacidae, Hemiptera) reveals insights into its “Jekyll and Hyde” mode of infection pattern. Genome Biol Evol 12:3818–3831. doi:10.1093/gbe/evaa006. 31958110 PMC7046167

[85] Kanonenberg K, Spitz O, Erenburg IN, Beer T, Schmitt L. 2018. Type I secretion system-it takes three and a substrate. FEMS Microbiol Lett 365:fny094. doi:10.1093/femsle/fny094. 29788124

[86] Gillespie JJ, Ammerman NC, Dreher-Lesnick SM, Rahman MS, Worley MJ, Setubal JC, Sobral BS, Azad AF. 2009. An anomalous type IV secretion system in *Rickettsia* is evolutionarily conserved. PLoS One 4:e4833. doi:10.1371/journal.pone.0004833. 19279686 PMC2653234

[87] Grohmann E, Christie PJ, Waksman G, Backert S. 2018. Type IV secretion in Gram-negative and Gram-positive bacteria. Mol Microbiol 107:455–471. doi:10.1111/mmi.13896. 29235173 PMC5796862

[88] Bhattacharya T, Newton ILG. 2019. Mi casa es su casa: how an intracellular symbiont manipulates host biology. Environ Microbiol 21:3188–3196. doi:10.1111/1462-2920.13964. PMC5924462 29076641

[89] Gillespie JJ, Phan IQH, Scheib H, Subramanian S, Edwards TE, Lehman SS, Piitulainen H, Rahman MS, Rennoll-Bankert KE, Staker BL, Taira S, Stacy R, Myler PJ, Azad AF, Pulliainen AT. 2015. Structural insight into how bacteria prevent interference between multiple divergent type IV secretion systems. mBio 6:e01867-15. doi:10.1128/mBio.01867-15. 26646013 PMC4676284

[90] Henderson KL, Felth LC, Molzahn CM, Shkel I, Wang S, Chhabra M, Ruff EF, Bieter L, Kraft JE, Record MT, Jr. 2017. Mechanism of transcription initiation and promoter escape by E. coli RNA polymerase. Proc Natl Acad Sci U S A 114:E3032–E3040. doi:10.1073/pnas.1618675114. 28348246 PMC5393250

[91] Lee J, Borukhov S. 2016. Bacterial RNA polymerase-DNA interaction-the driving force of gene expression and the target for drug action. Front Mol Biosci 3:73. doi:10.3389/fmolb.2016.00073. 27882317 PMC5101437

[92] Sutherland C, Murakami KS. 2018. An introduction to the structure and function of the catalytic core enzyme of *Escherichia coli* RNA polymerase. EcoSal Plus 8:ESP-0004-2018. doi:10.1128/ecosalplus.ESP-0004-2018. PMC6095464 30109846

[93] Kent BN, Bordenstein SR. 2010. Phage WO of *Wolbachia*: lambda of the endosymbiont world. Trends Microbiol 18:173–181. doi:10.1016/j.tim.2009.12.011. 20083406 PMC2862486

[94] Bordenstein SR, Bordenstein SR. 2016. Eukaryotic association module in phage WO genomes from *Wolbachia*. Nat Commun 7:13155. doi:10.1038/ncomms13155. 27727237 PMC5062602

[95] Iturbe-Ormaetxe I, Burke GR, Riegler M, Neill SL. 2005. Distribution, expression, and motif variability of ankyrin domain genes in *Wolbachia pipientis*. J Bacteriol 187:5136–5145. doi:10.1128/JB.187.15.5136-5145.2005. 16030207 PMC1196006

[96] Siozios S, Ioannidis P, Klasson L, Andersson SGE, Braig HR, Bourtzis K. 2013. The diversity and evolution of *Wolbachia* ankyrin repeat domain genes. PLoS One 8:e55390. doi:10.1371/journal.pone.0055390. 23390535 PMC3563639

[97] Walker T, Klasson L, Sebaihia M, Sanders MJ, Thomson NR, Parkhill J, Sinkins SP. 2007. Ankyrin repeat domain-encoding genes in the *w*Pip strain of *Wolbachia* from the *Culex pipiens* group. BMC Biol 5:39. doi:10.1186/1741-7007-5-39. 17883830 PMC2045654

[98] Cavanagh AT, Klocko AD, Liu X, Wassarman KM. 2008. Promoter specificity for 6S RNA regulation of transcription is determined by core promoter sequences and competition for region 4.2 of sigma70. Mol Microbiol 67:1242–1256. doi:10.1111/j.1365-2958.2008.06117.x. 18208528

[99] Schlüter JP, Reinkensmeier J, Barnett MJ, Lang C, Krol E, Giegerich R, Long SR, Becker A. 2013. Global mapping of transcription start sites and promoter motifs in the symbiotic α-proteobacterium *Sinorhizobium meliloti* 1021. BMC Genomics 14:156. doi:10.1186/1471-2164-14-156. 23497287 PMC3616915

[100] Trotochaud AE, Wassarman KM. 2005. A highly conserved 6S RNA structure is required for regulation of transcription. Nat Struct Mol Biol 12:313–319. doi:10.1038/nsmb917. 15793584

[101] Wassarman KM, Storz G. 2000. 6S RNA regulates *E. coli* RNA polymerase activity. Cell 101:613–623. doi:10.1016/s0092-8674(00)80873-9. 10892648

[102] Weinberg Z, Wang JX, Bogue J, Yang J, Corbino K, Moy RH, Breaker RR. 2010. Comparative genomics reveals 104 candidate structured RNAs from bacteria, archaea, and their metagenomes. Genome Biol 11:R31. doi:10.1186/gb-2010-11-3-r31. 20230605 PMC2864571

[103] Mayoral JG, Hussain M, Joubert DA, Iturbe-Ormaetxe I, O'Neill SL, Asgari S. 2014. *Wolbachia* small noncoding RNAs and their role in cross-kingdom communications. Proc Natl Acad Sci U S A 111:18721–18726. doi:10.1073/pnas.1420131112. 25512495 PMC4284532

[104] Fan C, Wu YH, Decker CM, Rohani R, Gesell Salazar M, Ye H, Cui Z, Schmidt F, Huang WE. 2019. Defensive function of transposable elements in bacteria. ACS Synth Biol 8:2141–2151. doi:10.1021/acssynbio.9b00218. 31375026

[105] Vigil-Stenman T, Ininbergs K, Bergman B, Ekman M. 2017. High abundance and expression of transposases in bacteria from the Baltic Sea. ISME J 11:2611–2623. doi:10.1038/ismej.2017.114. 28731472 PMC5649170

[106] Lindsey ARI, Bhattacharya T, Newton I, Hardy RW. 2021. *Wolbachia* and virus alter the host transcriptome at the interface of nucleotide metabolism pathways. mBio 12:e03472-20. doi:10.1128/mBio.03472-20. 33563832 PMC7885120

[107] Parry R, Bishop C, De Hayr L, Asgari S. 2019. Density-dependent enhanced replication of a densovirus in *Wolbachia*-infected *Aedes* cells is associated with production of piRNAs and higher virus-derived siRNAs. Virology 528:89–100. doi:10.1016/j.virol.2018.12.006. 30583288

[108] O'Neill SL, Pettigrew MM, Sinkins SP, Braig HR, Andreadis TG, Tesh RB. 1997. *In vitro* cultivation of *Wolbachia pipientis* in an *Aedes albopictus* cell line. Insect Mol Biol 6:33–39. doi:10.1046/j.1365-2583.1997.00157.x. 9013253

[109] Ridley AW, Hereward JP, Daglish GJ, Raghu S, McCulloch GA, Walter GH. 2016. Flight of *Rhyzopertha dominica* (Coleoptera: Bostrichidae)—a spatio-temporal analysis with pheromone trapping and population genetics. J Econ Entomol 109:2561–2571. doi:10.1093/jee/tow226. 27986943

[110] Pfaffl MW, Horgan GW, Dempfle L. 2002. Relative expression software tool (REST) for group-wise comparison and statistical analysis of relative expression results in real-time PCR. Nucleic Acids Res 30:e36. doi:10.1093/nar/30.9.e36. 11972351 PMC113859

[111] Asad S, Hussain M, Hugo L, Osei-Amo S, Zhang G, Watterson D, Asgari S. 2018. Suppression of the pelo protein by *Wolbachia* and its effect on dengue virus in *Aedes aegypti*. PLoS Negl Trop Dis 12:e0006405. doi:10.1371/journal.pntd.0006405. 29641562 PMC5912784

[112] Bishop C, Parry R, Asgari S. 2020. Effect of *Wolbachia w*AlbB on a positive-sense RNA negev-like virus: a novel virus persistently infecting *Aedes albopictus* mosquitoes and cells. J Gen Virol 101:216–225. doi:10.1099/jgv.0.001361. 31846415

[113] Robinson MD, Oshlack A. 2010. A scaling normalization method for differential expression analysis of RNA-seq data. Genome Biol 11:R25. doi:10.1186/gb-2010-11-3-r25. 20196867 PMC2864565

[114] McCarthy DJ, Chen Y, Smyth GK. 2012. Differential expression analysis of multifactor RNA-Seq experiments with respect to biological variation. Nucleic Acids Res 40:4288–4297. doi:10.1093/nar/gks042. 22287627 PMC3378882

[115] Ye J, Coulouris G, Zaretskaya I, Cutcutache I, Rozen S, Madden TL. 2012. Primer-BLAST: a tool to design target-specific primers for polymerase chain reaction. BMC Bioinformatics 13:134. doi:10.1186/1471-2105-13-134. 22708584 PMC3412702

