# Supplementary figures and images for: Transcriptional Response of Wolbachia to Dengue Virus Infection in Cells of the Mosquito Aedes aegypti
Source: mSphere. 2021 Jun 30;6(3):e00433-21. doi: 10.1128/mSphere.00433-21 (PMC8265661; doi:10.1128/mSphere.00433-21)

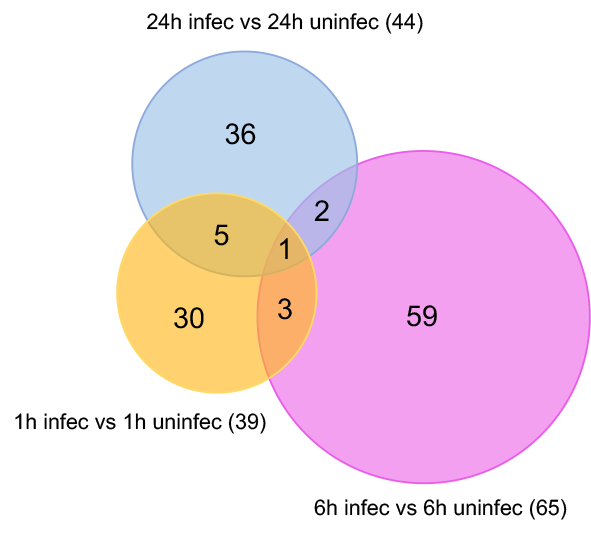

Supplement: FIG S2 [file msphere.00433-21-sf002.tif]

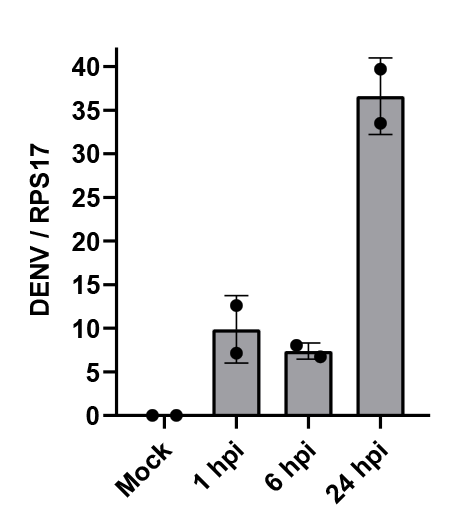

Supplement: FIG S3 [file msphere.00433-21-sf003.tif]

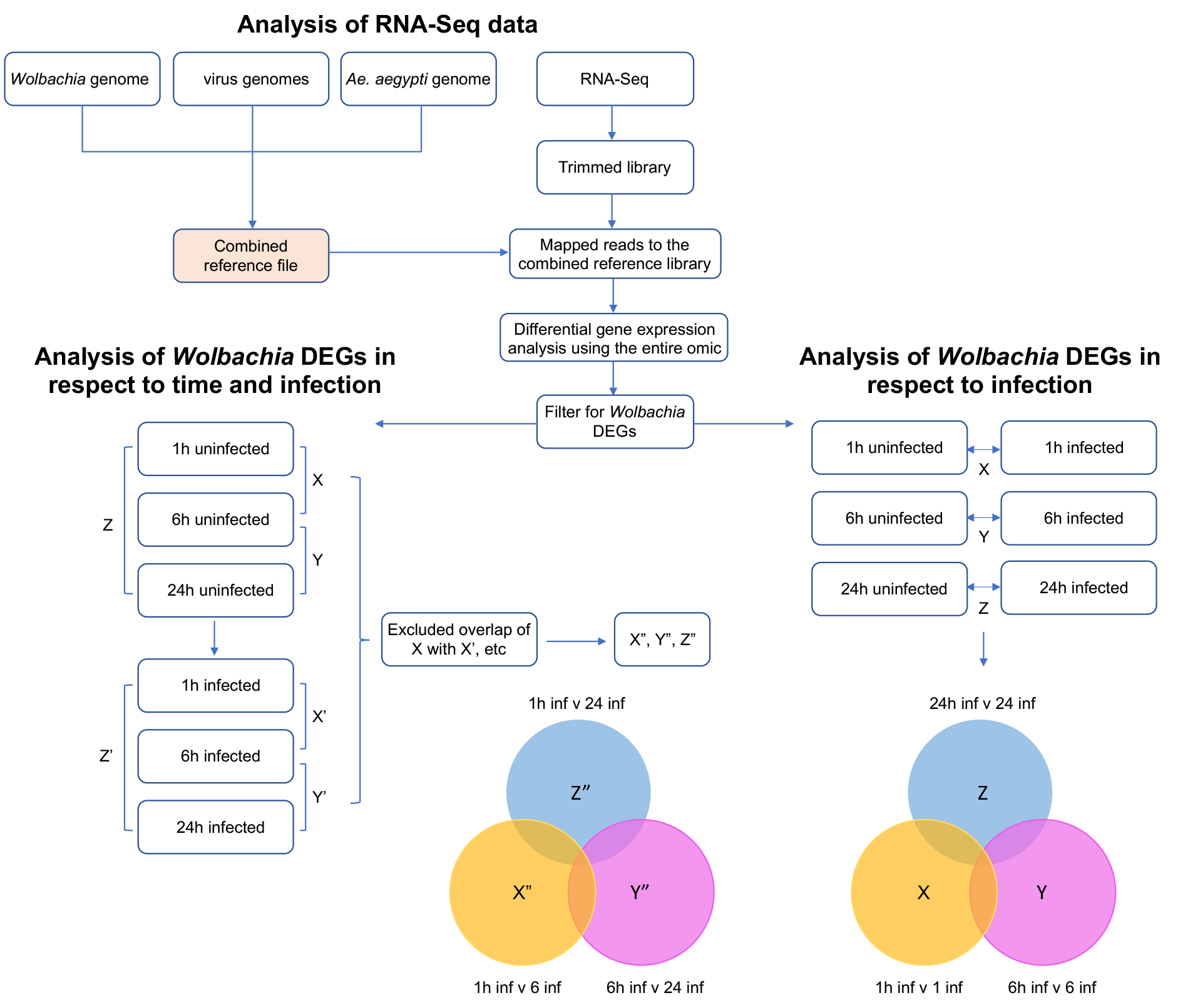

Supplement: FIG S1 [file msphere.00433-21-sf001.tif]
