# Supplementary material for: Transcriptional Response of Wolbachia to Dengue Virus Infection in Cells of the Mosquito Aedes aegypti
Source: mSphere. 2021 Jun 30;6(3):e00433-21. doi: 10.1128/mSphere.00433-21 (PMC8265661; doi:10.1128/mSphere.00433-21)
Supplement: TABLE S1 [file msphere.00433-21-st001.pdf]

Gene Name	Primer Sequence (5'-3')
wAlbB-wsp-qF	ATCTTTTATGGCTGGTGGTGCT
wAlbB-wsp-qR	GGAGTGATAGGCATATCTTCAAT
DENV2-qF	GGTATGGTGGGCGCTACTA
DENV2-qR	CAAGGCTAACGCATCAGTCA
AeRPS17-qF	CACTCCGAGGTCCGTGGTAT
AeRPS17-qR	GGACACTTCGGGCACGTAGT
DnaJ-04285-qF	AAGAAATGCGGTGGAAGTGG
DnaJ-04285-qR	TGCTCCAGCTTCTCCTTTACC
AtpBC-00395-qF	ACGCCTAGCAATCGAGAAGT
AtpBC-00395-qR	GCAAGTGCCACTCTTTTCTTCA
RNaseH-03730-qF	TGTGCTTTTACCCACTTCCAAT
RNaseH-03730-qR	TGTGCTTTTACCCACTTCCAAT
OMT-02160-qF	TGAATTATCAGCAAAGGCACCA
OMT-02160-qR	AATGTATGACTCCGCCCAATCT
FeScl-04465-qF	AAGCAAAAAGGATGCTCTGGTT
FeScl-04465-qR	TCGATCAGCACCTTGACTTTT
16SrRNA-05705-qF	GAGCGCAACCCTCATCCTTA
16SrRNA-05705-qR	ATCATCCCCACCTTCCTCCA
GrpE-05955-qF	GGTCAGCAAACGAAAAGGGGA
GrpE-05955-qR	GCAACAGCGCGACGTAAATG
